# Mutations in the nucleotide binding and hydrolysis domains of *Helicobacter pylori* MutS2 lead to altered biochemical activities and inactivation of its *in vivo* function

**DOI:** 10.1186/s12866-016-0629-3

**Published:** 2016-02-03

**Authors:** Prashant P. Damke, Rajkumar Dhanaraju, Stéphanie Marsin, J. Pablo Radicella, Desirazu N. Rao

**Affiliations:** Department of Biochemistry, Indian Institute of Science, Bangalore, 560012 India; CEA, Institute of Cellular and Molecular Radiobiology, Fontenay aux Roses, France; INSERM UMR967, Fontenay aux Roses, France; Universités Paris Diderot et Paris Sud, Fontenay aux Roses, France

**Keywords:** *Helicobacter pylori*, Transformation, MutS2, ATPase, DNA binding, Nuclease

## Abstract

**Background:**

*Helicobacter pylori* MutS2 (HpMutS2), an inhibitor of recombination during transformation is a non-specific nuclease with two catalytic sites, both of which are essential for its anti-recombinase activity. Although HpMutS2 belongs to a highly conserved family of ABC transporter ATPases, the role of its ATP binding and hydrolysis activities remains elusive.

**Results:**

To explore the putative role of ATP binding and hydrolysis activities of HpMutS2 we specifically generated point mutations in the nucleotide-binding Walker-A (HpMutS2-G338R) and hydrolysis Walker-B (HpMutS2-E413A) domains of the protein. Compared to wild-type protein, HpMutS2-G338R exhibited ~2.5-fold lower affinity for both ATP and ADP while ATP hydrolysis was reduced by ~3-fold. Nucleotide binding efficiencies of HpMutS2-E413A were not significantly altered; however the ATP hydrolysis was reduced by ~10-fold. Although mutations in the Walker-A and Walker-B motifs of HpMutS2 only partially reduced its ability to bind and hydrolyze ATP, we demonstrate that these mutants not only exhibited alterations in the conformation, DNA binding and nuclease activities of the protein but failed to complement the hyper-recombinant phenotype displayed by *mutS2-*disrupted strain of *H. pylori*. In addition, we show that the nucleotide cofactor modulates the conformation, DNA binding and nuclease activities of HpMutS2.

**Conclusions:**

These data describe a strong crosstalk between the ATPase, DNA binding, and nuclease activities of HpMutS2. Furthermore these data show that both, ATP binding and hydrolysis activities of HpMutS2 are essential for the *in vivo* anti-recombinase function of the protein.

**Electronic supplementary material:**

The online version of this article (doi:10.1186/s12866-016-0629-3) contains supplementary material, which is available to authorized users.

## Background

Natural transformation (NT) is an important route to generate genetic and phenotypic diversity, which is a prerequisite for evolution and adaptation of *Helicobacter pylori* in the dynamic gastric niche of human hosts. In the naturally and constitutively competent *H. pylori,* incorporation of exogenous DNA into the genome through recombination plays a major role in the acquisition and spread of antigenic variants and drug resistance genes. Numerous studies have demonstrated that mixed infections by *H. pylori* promote persistence of the infection and disease progression [1, 2]. Although some aspects of the processes that determine the successful uptake and incorporation of exogenous DNA in *H. pylori* have been addressed [3, 4], the mechanisms that limit inter-genomic recombination are still poorly understood. Several studies in *H. pylori* and other bacteria have demonstrated that MutS2 limits transformation through nucleolytic cleavage of DNA intermediates of the homologous recombination (HR) pathway [5–7]. Contrary to other studied MutS2 proteins, *H. pylori* MutS2 (HpMutS2) possesses an additional nuclease site at its N-terminus (LDLK) in addition to the canonical C-terminal Smr domain. *In vivo* studies showed that the presence of both nuclease sites is essential to secure the anti-recombinase function of the protein [7].

The main similarity of MutS2 proteins with other MutS paralogues relies on the ATPase and dimerization region. In fact, the classification of MutS homologues was based primarily on the similarity found in this region [5, 8–10]. MutS2 belongs to the ATP-binding cassette (ABC) transporter family of ATPases. It contains Walker-A (GxGKS/T) and Walker-B motifs (DExx) characteristic of proteins that bind and hydrolyze NTP, respectively [10]. Nucleotide cofactors play a critical role in regulation of structure and function of several proteins to which they bind. Notably, in the case of the distant paralogue of MutS2, MutS1, the mismatch binding protein uses ATP to regulate DNA-protein and protein-protein interactions. The binding of ATP after mismatch recognition by MutS1 initiates a cascade of reactions resulting in the repair of the mismatch [11–15]. Moreover, the functions of eukaryotic MutS homologues (MSH proteins) are also modulated by ATP binding and hydrolysis [16–20]. Several studies reported that mutations in the nucleotide binding domains of MutS homologues often resulted in the failure to initiate the MMR pathway [17, 19]. Communication between the ATPase activity and mismatch recognition domains of MutS homologues is mediated by conformational changes induced in the protein. It has been shown that binding of ATP to MutS induces conformational changes in other regions of the protein complexes [21]. Since bacterial MutS1 and MutS2 proteins show substantial conservation in their ATPase domains, it is tempting to propose that HpMutS2 activities could be influenced by binding of ATP and other nucleotide cofactors. However, the correlations between the DNA binding, nuclease and ATPase activities of HpMutS2 have not been thoroughly analyzed yet. In this study, using wild-type and mutant forms of HpMutS2 defective in ATP binding or hydrolysis, the influence of ATP and other nucleotides binding and hydrolysis was monitored on the structure and function of HpMutS2. We observed that ATP modulates the conformation, DNA binding and nuclease activities of HpMutS2. Interestingly, mutations in the critical amino acids residues abrogating the nucleotide binding and hydrolysis resulted in loss of anti-recombinase activity of the protein in the cells.

## Results and discussion

### Construction and purification of HpMutS2 and variants

**Fig. 1:**
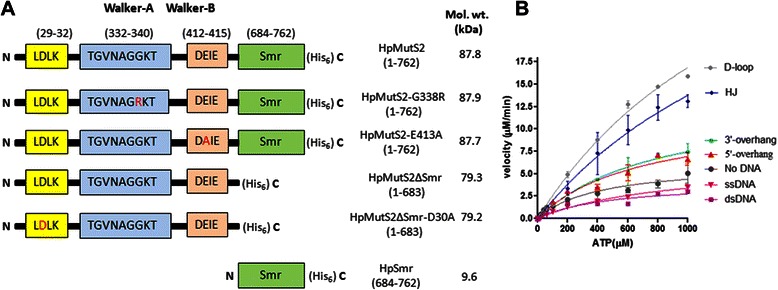
**a** Schematic representation of HpMutS2 variants used in this study. Walker-A and Walker-B motifs of HpMutS2 were identified by multiple sequence alignment of MutS2 proteins from different bacteria using Clustal Omega (http://www.ebi.ac.uk/Tools/msa/clustalo/). The mutations in the walker-A and Walker-B motifs were introduced using site directed mutagenesis. The mutated amino acids are highlighted in red. All the proteins were C-terminally His_6_-tagged. The LDLK motif and Smr domain are conserved nuclease sites of HpMutS2. **b** Effect of DNA substrates on ATPase activity of HpMutS2. HpMutS2 (45 nM) was incubated with increasing concentrations of ATP [0, 10, 20, 40, 60, 80, 100, 200, 400, 600, 800, and 1000 (μM)]. DNA substrates (1 μM) were added separately to the reaction mixtures. After incubation at 37 °C for 30 min the reactions were stopped by EDTA (50 mM) and the products were separated by TLC. ATP [γ-^32^P] was used as tracer to monitor the product formation. Reaction velocities were calculated by quantifying the proportion of products formed to un-reacted substrate divided by incubation time

Multiple sequence alignment of MutS2 proteins from different bacteria revealed that HpMutS2 possesses highly conserved Walker-A motif: TGVNAGGKT (amino acids 332–340) and Walker-B motif: DEIE (amino acids 412–415) known to mediate binding and hydrolysis of ATP respectively (Additional file 1: Figure S1). In an earlier study it was shown that mutation in the conserved Gly (G338R) of Walker-A motif resulted in loss of ATP hydrolysing activity of HpMutS2 [5]. In case of *Thermus aquaticus* MutS, mutations in the conserved Glu residue of Walker-B motif resulted in ~100-fold loss of ATP hydrolysing activity [22]. Therefore, to confirm the putative roles of these amino acids of Walker-A and Walker-B motifs of HpMutS2 and to delineate the possible role(s) of the nucleotide binding and hydrolysis activities of the protein, we specifically generated point mutations in the Walker-A (HpMutS2-G338R) and Walker-B (HpMutS2-E413A) motifs of HpMutS2 by site directed mutagenesis. The schematic representation of mutants and other variants of HpMutS2 used in this study are displayed in Fig. 1a. The His_6_-tagged proteins over-expressed in *Escherichia coli* were purified as described in the Methods section. The homogeneity of the purified proteins was judged by SDS-polyacrylamide gel electrophoresis (data not shown).

### ATP binding/hydrolysis by HpMutS2, HpMutS2-G338R, and HpMutS2-E413A

To delineate the characteristics of the ATPase activity of HpMutS2, we initially determined the metal ion requirement for ATP hydrolysis by HpMutS2. Purified HpMutS2 was incubated with excess of cold ATP (100 μM) with or without metal ions (5 mM each), and ATP hydrolysis monitored by thin layer chromatography using radiolabeled ATP [γ-^32^P] as tracer. Additional file 1: Figure S2A shows that no ATP hydrolysis was observed in the absence of metal ion or when metal ions were incubated with the ATP alone. However, the presence of Mg^2+^ supported a strong ATPase activity. EDTA (50 mM) inhibited the Mg^2+^ dependent ATPase activity indicating that HpMutS2 is a metal dependent ATPase (Additional file 1: Figure S2A lane 18). Albeit with less efficiency, Mn^2+^, Cd ^2+^ Co^2+^, Cu^2+^, Ca^2+^ or Zn^2+^ (5 mM each) also allowed the ATPase activity to various extents. Interestingly, Ni^2+^, particularly abundant in *H. pylori,* did not significantly support the ATPase activity (Additional file 1: Figure S2A, lane 11). A time dependent hydrolysis of ATP demonstrated that the initial rates of ATP hydrolysis were approximately linear up to 60 min (Additional file 1: Figure S2B). When increasing concentrations of HpMutS2 were incubated with excess of ATP, it exhibited concentration-dependent hydrolysis of the nucleotide (Additional file 1: Figure S2C). HpMutS2ΔSmr, a truncated version of HpMutS2 lacking the C-terminal Smr domain, exhibited similar rates of ATP hydrolysis compared to the wild-type protein. The C-terminal nuclease domain of HpMutS2, HpSmr, by itself did not have ATP hydrolysis activity (Additional file 1: Figure S2C). Taken together these results suggest that the Smr domain of HpMutS2 does not contribute to the ATPase activity of the protein. Based on the above observations, all subsequent ATPase assays were performed in presence of Mg^2+^ (5 mM) at 37 °C using the protein concentrations in the linear range of enzymatic activity for 30 min or below.

To further characterize the ATPase activity of HpMutS2, initial velocity of the ATP hydrolysis reaction was analysed as a function of ATP concentration. Non-linear regression analysis of the plot yielded a *K*_m_ for ATP of 412.1 ± 83.43 μM, while the *k*_cat_ value was 41.77 ± 2.88 min^−1^(Table 1). Compared to *Thermus thermophilus* MutS2 (TtMutS2) [23], the *K*_m_ value obtained for HpMutS2 is ~9-fold higher while the *k*_cat_ value is ~20-fold higher. Compared to *T. thermophilus* MutS1 [15] the *K*_*m*_ values are ~8-fold higher while the *k*_cat_ values are ~4.5-fold higher. Next, to monitor the effect of DNA on ATPase activity of HpMutS2 various DNA substrates (1 μM) mimicking HR intermediates were added separately to the reaction mixture containing HpMutS2 and increasing concentrations of ATP (0–1 mM). As can be seen in Fig. 1b, the ATPase activity of HpMutS2 was stimulated in presence of branched DNA substrates such as Holliday junction and D-loop by ~4-fold whereas no effect was observed with linear DNA substrates such as ssDNA, dsDNA, and only negligible increases with 3′- or 5′-overhangs. Similar results were reported previously for HpMutS2 [5] and *Thermotoga maritima* MutS2 (TmMutS2) [24]. MutS homologues involved in mismatch repair, such as bacterial MutS1 or the hMSH2-hMSH6 complex, exhibit similar conversion of ATP to ADP, however they require the presence of mismatched DNA [20, 25, 26]. On the other hand, eucaryotic hMSH4-hMSH5, implicated in meiotic recombination [27], shows stimulation of its ATPase activity in presence of Holliday junction [28].

**Table 1:** Kinetic parameters for ATPase activities of HpMutS2 and mutants

	*K*_*m*_ (μM)	*V*_max_ (μM min^−1^)	*k*_cat_ (min^−1^)
HpMutS2	412.1 ± 83.43	1.88 ± 0.13	41.77 ± 2.88
HpMutS2-G338R	939.3 ± 489.2	0.64 ± 0.15	14.22 ± 3.33
HpMutS2-E413A	373.2 ± 188.6	0.19 ± 0.03	4.22 ± 0.66

The kinetic parameters for HpMutS2, HpMutS2-G338R, and HpMutS2-E413A were determined by varying the substrate concentration and performing Michaelis-Menten analysis. The error values represent standard deviation from at least two different experiments

**Fig. 2:**
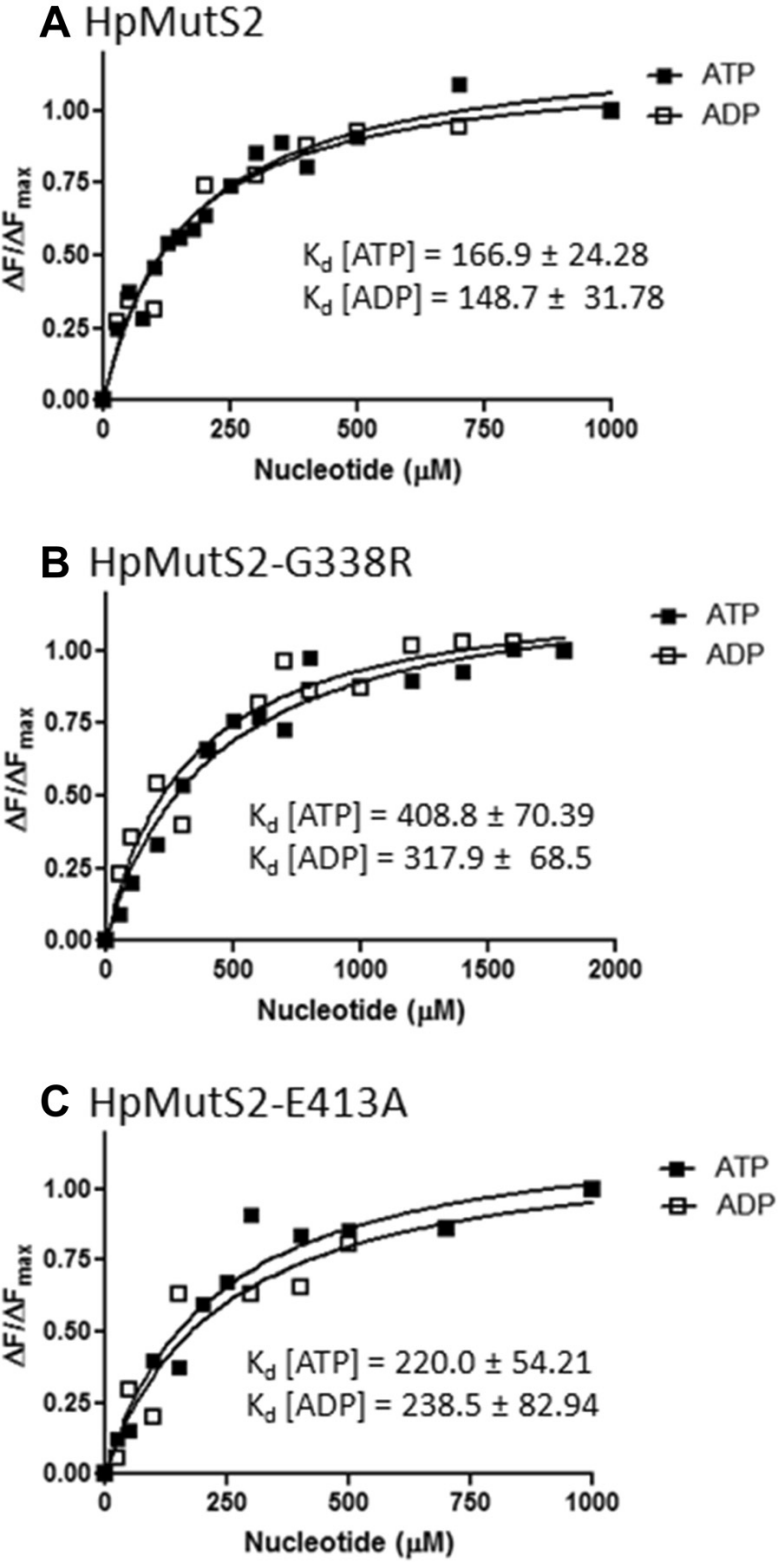
Nucleotide binding affinities of **a** HpMutS2 **b** HpMutS2-G338R and **c** HpMutS2-E413A. Tryptophan fluorescence assays described in the Methods section were used to determine the nucleotide binding affinities of proteins. The dissociation constants were calculated by non-linear regression analysis of plots of ΔF/ΔF_max_ versus the ligand concentration. ΔF: magnitude of the difference between the observed fluorescence intensity at a given ligand concentration and the fluorescence intensity in the absence of any ligand, ΔF_max_: difference at an infinite ligand concentration

Next, the ATP hydrolysing abilities of HpMutS2-G338R and HpMutS2-E413A were tested and compared to that of wild-type HpMutS2. Table 1 shows that compared to the wild-type protein, HpMutS2-G338R exhibits ~2.3-fold higher *K*_m_ value indicating reduced affinity for ATP and reduced (~3-fold) *k*_cat_ value. HpMutS2-E413A shows a *K*_m_ value comparable to that of the wild-type protein, however it exhibits ~10-fold reduced *k*_cat_ value. The residual ATP hydrolysis activity in the case of the HpMutS2-G338R mutant (Table 1) suggests that this mutant protein might still be able to bind ATP, albeit much less efficiently. Therefore, to determine the nucleotide binding efficiencies of HpMutS2, HpMutS2-G338R, and HpMutS2-E413A, intrinsic fluorescence properties of these proteins were exploited. HpMutS2 has two tryptophan residues at positions 551 and 644. A fluorescence-quenching assay was used to determine the ATP and ADP binding affinities of HpMutS2 and its mutants. Quenching of the Trp fluorescence was recorded upon titration with ATP and ADP in absence of any divalent metal ions. Saturation isotherms generated from the changes in the fluorescence emission spectra of HpMutS2, HpMutS2-G338R, and HpMutS2-E413A were used to calculate the dissociation constants for ATP and ADP (Figs. 2a, b and c respectively). The K_d_ value obtained for ATP for wild-type HpMutS2 (166.9 ± 24.28 μM) is ~2.4-fold less to its *K*_m_ value (Table 1). No significant differences in binding affinities of the wild-type protein for ATP and ADP (148.7 ± 31.78 μM) were observed (Fig. 2a). Compared to the wild-type protein, the HpMutS2-G338R mutant showed ~2.5 and 2.2-fold reduced affinities whereas the HpMutS2-E413A mutant showed negligible 1.3 and 1.6-fold reduced affinities for ATP and ADP, respectively (compare Figs. 2a, b, and c). ATP hydrolysis and binding assays confirm that Gly-338 (Walker-A) is partially responsible for nucleotide binding while Glu-413 (Walker-B) is required for hydrolysis. When compared to other MutS paralogues, the K_d_ value obtained for the wild-type HpMutS2 are 5–40-fold lower [16, 28–30]. Moreover, the nucleotide binding assays were performed in the absence of divalent cations, indicating that HpMutS2 does not require any divalent cation for nucleotide binding.

### Nucleotide and mutations induced conformational transitions in HpMutS2

**Fig. 3:**
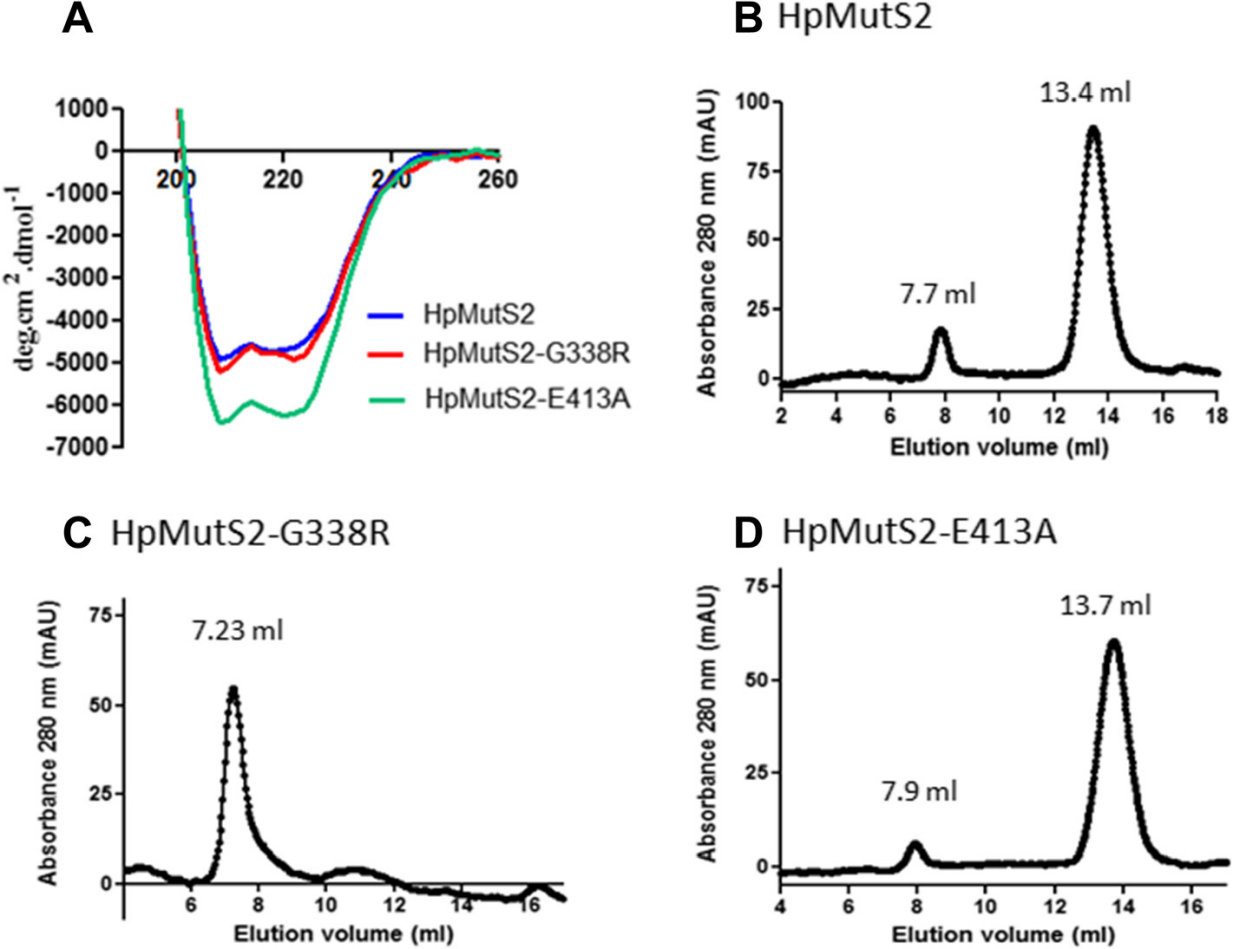
Mutation induced conformational changes in HpMutS2. **a** CD-spectroscopy of HpMutS2, HpMutS2-G338R, and HpMutS2-E413A**.** Far-UV CD spectra of proteins (1 μM) dialyzed in 50 mM Tris-Cl and 100 mM NaCl were recorded between 200–300 nm with 20 mdeg sensitivity at a scan speed of 50 nm using 2 mm path length cuvette with a bandwidth of 2 nm. The spectra were recorded using Jasco-815 spectropolarimeter. Size exclusion chromatographic analysis of **b** HpMutS2, **c** HpMutS2-G338R, and **d** HpMutS2-E413A was performed using Superose-6 gel filtration column at a constant flow rate of 0.3 ml/min. Approximately 150 μg of all the proteins were spun at 10,000 rpm for 10 min at 4 °C before injection. The absorbance was recorded at 280 nm

**Fig. 4:**
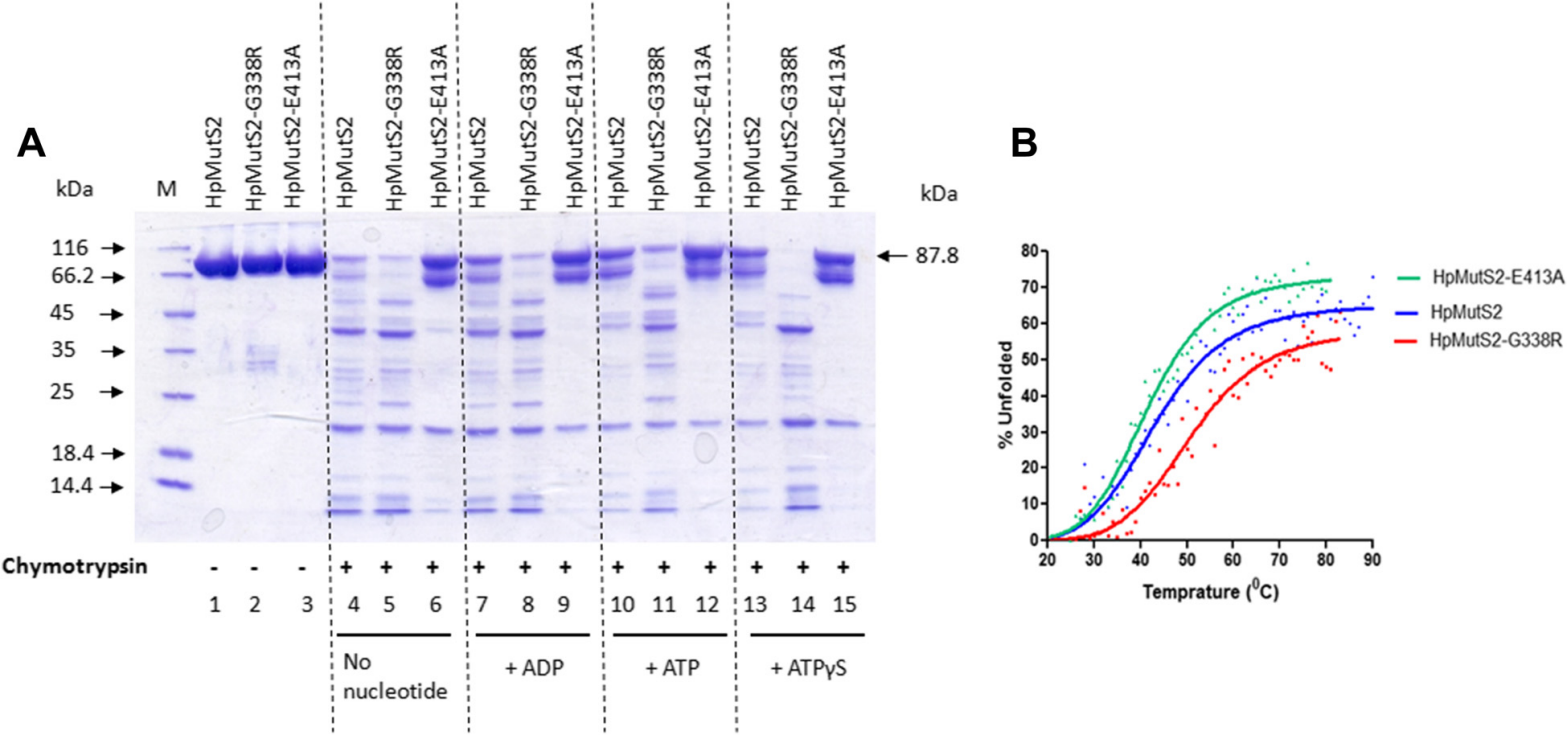
Nucleotide and mutation induced conformational changes in HpMutS2. **a** Limited proteolysis. Proteins (4 μM) either free or pre-incubated with nucleotides (1 mM) were incubated with chymotrypsin (1.25 ng) for 30 min at 37 °C. All proteolysis reactions were performed in 1X buffer A lacking magnesium. Reactions were stopped by adding a protease inhibitor cocktail and the products were separated on SDS-PAGE (10 %). Lane M: Molecular weight marker. **b** Thermal stability profiles of wild-type HpMutS2, HpMutS2-G338R, and HpMutS2-E413A. Proteins (1 μM) dialyzed in 50 mM Tris-Cl and 100 mM NaCl were subjected to thermal denaturation and spectra were recorded at 222 nm using Jasco-815 spectropolarimeter

Several studies have reported that the conformation of MutS proteins is altered by the presence of ATP or ADP and due to mutations in the nucleotide binding and hydrolysis domains [11–14, 21, 25, 31]. To examine such possibility in HpMutS2, circular dichroism (CD) spectroscopy was employed to detect gross alterations in the secondary structures of HpMutS2 and mutant proteins. Fig. 3a shows that two minima were observed at 222 nm and 209 nm which suggest a high degree of α-helical structure in HpMutS2. Interestingly, compared to the wild-type protein no significant changes were observed in HpMutS2-G338R while HpMutS2-E413A exhibited ~30–34 (%) reduced negative ellipticity (Fig. 3a). We next performed gel filtration analysis to monitor the effect of the mutations on the tertiary structures of the proteins. Wild-type HpMutS2 eluted from a superpose-6 column as a major peak with absorbance maxima at 13.4, the molecular mass estimated using a standard graph (data not shown) was ~307 kDa corresponding to a tetramer (Fig. 3b). The tetrameric form of HpMutS2 was validated in a previous study using gel filtration and dynamic light scattering experiments [7]. A small fraction of the wild-type protein eluted at 7.7 ml, close to the void volume of the column (Fig. 3b) with molecular mass of ~20067 kDa indicating formation of multimers by the protein. Compared to the wild-type protein, a slight increase in the elution volume of HpMutS2-E413A was observed (compare Figs. 3b and d). HpMutS2-E413A eluted as a major peak (13.7 ml) with molecular mass of ~254 kDa and a minor peak (7.9 ml) with molecular mass of ~17338 kDa. Interestingly, HpMutS2-G338R eluted as a single major peak at 7.23 ml, close to the void volume with molecular mass of ~ 28291 kDa, with no apparent peak corresponding to a tetramer (Fig. 3c). We observed similar behaviour of this mutant even at half the concentration of the protein (data not shown). In order to substantiate conformational changes in HpMutS2 due to mutations in the nucleotide binding and hydrolysis domains, limited proteolysis was performed using chymotrypsin that has close to 205 cleavage sites in this protein. Fig. 4a shows that the proteolysis pattern obtained differed significantly for the wild-type and mutant proteins. As evident by the number and density of resulting bands, HpMutS2-G338R is slightly more susceptible to proteolysis compared to the wild-type protein, whereas HpMutS2-E413A is much more resistant (Fig. 4a, lanes 4–6). To detect nucleotide induced conformational transitions, the proteins were pre-incubated separately with ATP or ADP (1 mM each) for 15 min at 37 °C before addition of the chymotrypsin. ATPγS was also used to rule out any residual activity due to metal contamination. It can be seen that while pre-incubation with ADP, ATP or ATPγS had only a slight effect on the degradation patterns of HpMutS2-G338R (Fig. 4a, lanes 8, 11 and 14, respectively) and HpMutS2-E413A (Fig. 4a, lanes 9, 12 and 15, respectively), proteolysis was significantly reduced in the case of the wild-type protein (Fig. 4a, lanes 7, 10, and 13 respectively). In general, it can be seen that the wild-type protein become more chymotrypsin resistant in the presence of ATP and ATPγS with a digestion pattern similar to the HpMutS2-E413A mutant. Additionally it can be seen that the protease resistance is more prominent in the case of ATP and its non-hydrolysable analogue (ATPγS) compared to ADP. At higher concentrations of chymotrypsin the conformational dissimilarities in the wild-type and mutant proteins were more apparent (Additional file 1: Figure S3, lanes 4, 5 and 6) while when the proteins were subjected to proteolysis after heat denaturation the patterns obtained were similar in all the cases (Additional file 1: Figure S3, lanes 11, 12 and 13). A thermal stability analyses performed using CD-spectroscopy demonstrated that the inherent stability of the HpMutS2 and variants differed significantly (Fig. 4b). 50 % unfolding of wild-type protein and HpMutS2-E413A were achieved at ~50 °C although at higher temperatures HpMutS2-E413A was more unstable. Consistent with the formation of higher multimeric complexes by HpMutS2-G338R, this mutant protein was more stable at up to 80 °C when compared to the wild-type and HpMutS2-E413A (Fig. 4b). Taken together, CD-spectroscopy, gel filtration and partial proteolysis suggest that mutations in the nucleotide binding and hydrolysis domains and interaction with nucleotides induce domain rearrangements in HpMutS2. Partial proteolysis experiments suggest that incubation of wild-type HpMutS2 with nucleotides results in a compact structure resistant to partial proteolysis. Consistently, HpMutS2-E413A, which should retain ATP bound due to mutation in the Walker-B motif, adopts a conformation more resistant to proteolysis. On the other hand, the HpMutS2-G338R is more sensitive to proteolysis indicating formation of a more open conformation. In the case of MutS1, the nucleotide binding motifs are located at the dimer-interface region of the protein [32], it is possible that in case of HpMutS2 the binding of ATP may favour a particular oligomeric state of the protein. HpMutS2-G338R multimers could be a consequence of its low affinity towards nucleotides as shown by fluorescence quenching assays (Fig. 2b). These results suggest that binding of ATP or ADP induces the formation of a compact structure while in the absence of nucleotides HpMutS2 adopts a more open conformation. Similar nucleotide induced compaction of the TmMutS2 was shown by limited proteolysis, gel filtration and SAXS analysis [24]. The large structural distortion observed in the case of HpMutS2-G338R and HpMutS2-E413A and the variation in thermal denaturation profiles indicated that the intact nucleotide binding and hydrolysis domains are essential to maintain the structural integrity of the protein.

### Effect of nucleotide cofactors on DNA binding and nuclease activities of HpMutS2

DNA binding and ATPase activities are essential for the function of MutS homologues *in vivo* [14, 33]. MutS1 dissociates from homoduplex or heteroduplex DNA upon interaction with ATP [11, 12, 34]. Even in the case of Msh2-6, distant relative of MutS2, ATP binding lowers the affinity for DNA [16]. Moreover, MutS1 uses ATP for translocation along DNA in search of a mismatch and for interaction with MutL [14, 35]. In an earlier study [7] we demonstrated that HpMutS2 exhibits sequence and structure non-specific DNA binding and nuclease activities. ATPase assays performed in this study showed that the ATP hydrolysis by HpMutS2 was enhanced by branched DNA substrate such as Holliday junction, whereas no significant effect of ssDNA was observed (Fig. 1b). During natural transformation the internalized ssDNA initiates the HR pathway. Based on studies in other bacteria [36], it is proposed that in *H. pylori,* RecA aided by DprA polymerizes on ssDNA and catalyses strand invasion and pairing of the donor DNA with the homologous chromosomal sequences [37]. The displacement loop (D-loop) thus generated is resolved into recombined DNA molecules. Here we used ssDNA and Holliday junction substrates to monitor the effect of nucleotides on DNA binding and nuclease activities of HpMutS2.

**Fig. 5:**
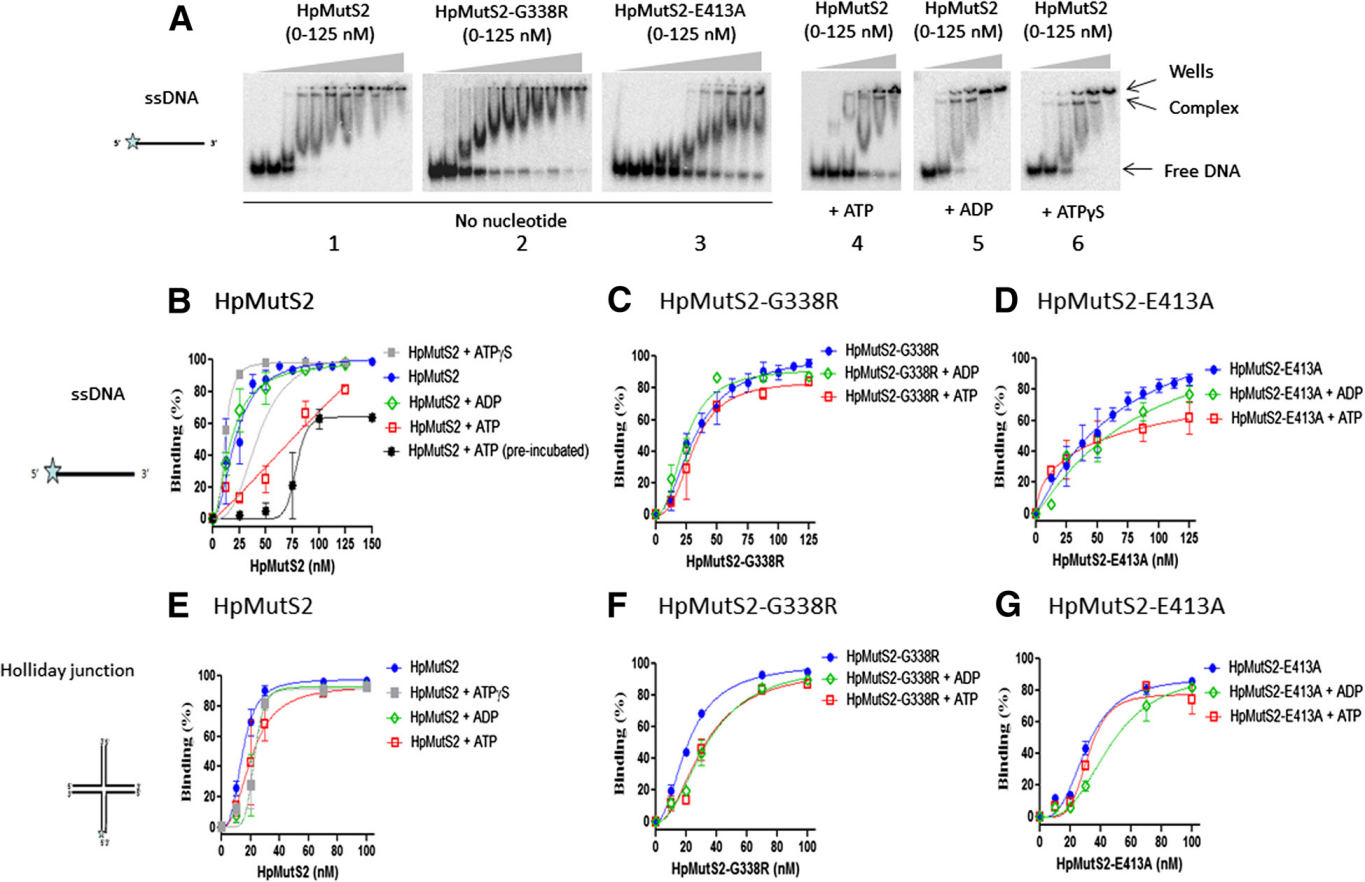
Effect of nucleotide cofactors on DNA binding activity of HpMutS2 and mutants. Electrophoretic mobility shift assays were performed by incubating radiolabeled DNA substrates (0.16 nM) with increasing concentrations of proteins as described in Methods section. Nucleotides (1 mM) were added separately to the reaction mixtures. After incubation of 30 min on ice, the reaction mixtures were electrophoresed on native PAGE (8 %) at 4 °C. (**a**, panel 1–6) Interaction of HpMutS2 and mutants with single-stranded DNA. **b**-**g** Plots of percentage of bound DNA substrate (ssDNA and Holliday junction) versus protein concentration. The percentage binding was calculated by measuring the depletion in substrate DNA considering DNA without protein as 100 %. Error bars represent standard deviation from two or more different experiments. Schematic representation of DNA substrates used are shown at the left side of figures

As expected [5, 7], gel retardation assays demonstrated that HpMutS2 forms a stable complex with ssDNA (Fig. 5a, panel 1) and with Holliday junctions (data not shown). The binding affinities calculated using a plot of bound DNA versus the protein concentrations were comparable (Figs. 5b, e, and Table 2). HpMutS2-G338R exhibited slightly reduced (~1.5-fold) affinity towards both ssDNA (Fig. 5a, panel 2) and Holliday junction (Fig. 5c, f, and Table 2) substrates. On the other hand, while HpMutS2-E413A exhibited ~2-fold reduced affinity on Holliday junction, ssDNA binding (Figs. 5a, panel 3) was reduced by > 4-fold (Figs. 5d, g, and Table 2). To detect the effect of nucleotides on the DNA binding capabilities of wild-type protein, different nucleotides (1 mM) were added to the reaction mixtures separately. At lower concentrations of the wild-type protein it exhibited ~5-fold reduced ssDNA binding, however, at higher concentrations no apparent change in the ssDNA binding was observed (Fig. 5a, panel 4, b, and Table 2). ADP and ATPγS had no significant effects on the ssDNA binding affinities of the wild-type protein (Fig. 5a, panel 5, 6, b, and Table 2). Interestingly, the presence of nucleotides did not affect Holliday junction binding by the wild-type protein (Fig. 5e and Table 2) and only minor effects of the nucleotides were observed on HpMutS2-G338R and HpMutS2-E413A mutants’ binding to either DNA substrate (Fig. 5c, d, f, G and Table 2). With ssDNA smearing of radioactivity could represent formation of several nucleoprotein complexes or the dissociation of the complexes during electrophoresis. A second band is observed in all the cases except for HpMutS2-G338R mutant. The topmost band probably represents larger nucleoprotein complex while the band below probably represents ssDNA bound to a protein tetramer. The absence of the second band in the case of the HpMutS2-G338R could be attributed to its ability to form only higher oligomeric species without formation of any tetramers or lower order multimers (Fig. 3c). In the case of HpMutS2 binding to ssDNA in presence of ATP (Fig. 5a, panel 4 and b) and that of HpMutS2-E413A binding to ssDNA (Fig. 5a panel 3 and d), the plot of bound DNA versus the concentration of protein did not reach a plateau even at higher concentrations of proteins (Figs. 5a, panel 3, d). Therefore, the dissociation constant and error values cannot be accurately calculated. However, the presence of ATP and the disruption of the ATP hydrolysing activity of HpMutS2 possibly lower the ssDNA binding affinity of HpMutS2 by ~4–5-fold without significantly affecting the Holliday junction binding activity (Fig. 5 and Table 2). It is tempting to propose that conformational states of HpMutS2 induced by the interaction with ATP reduce the inherent ability of the protein to bind ssDNA. Supporting this hypothesis, we observed that HpMutS2-E413A, which can bind but does not hydrolyse ATP, showed > 4-fold reduction in ssDNA binding capacity (Fig. 5d and Table 2). Similarly, ATP specific reduction of DNA binding affinity was observed with other linear DNA substrates such as dsDNA and 3′ or 5′-overhang containing DNA (Additional file 1: Figure S4A-C). On the other hand, as observed for the Holliday junction, the presence of ATP showed no significant alteration of the DNA binding capabilities of the wild-type protein on other branched DNA substrates such as D-loop and splayed duplex (Additional file 1: Figure S4D and E). These observations suggest that ATP decreases the DNA binding ability of HpMutS2 on linear DNA substrates. When wild-type HpMutS2 was pre-incubated with ATP prior to addition of ssDNA, a similar reduction in the DNA binding of HpMutS2 was observed (Fig. 5b). On the other hand, when a preformed ssDNA-HpMutS2 complex was exposed to increasing concentrations of ATP (0.25–5 mM) only ~20 % dissociation of ssDNA was observed at the highest concentration of ATP used (Additional file 1: Figure S5A), while presence of low concentrations of unlabelled ssDNA (500 nM) resulted in ~70 % displacement of the labelled ssDNA from the complex (Additional file 1: Figure S5B). These observations suggest that ATP, while decreasing HpMutS2 affinity for ssDNA, may not favour the dissociation of the protein from the preformed nucleoprotein complex. Similar results were obtained when a closed circular pUC19 DNA-HpMutS2 complex was incubated with increasing concentrations of ATP (Additional file 1: Figure S5C). In the case of MutS homologues only binding of the ATP is sufficient to induce the release of mismatch DNA whereas ATP hydrolysis is not required [38–41]. We performed all DNA binding experiments under non hydrolysing conditions. However, only ATP and not ADP or ATPγS show reduction in linear DNA substrate binding. It is possible that a particular conformational state influenced by ATP may favour the inherent ability of the protein to bind ssDNA and other linear DNA substrates.

**Table 2:** DNA binding affinities (nM) determined for HpMutS2 and mutants

	No nucleotide	+ ATP	+ ADP	+ ATPγS
Single-stranded DNA				
HpMutS2	21.48 ± 2.28	^a^	17.27 ± 2.89	11.44 ± 0.19
HpMutS2-G338R	32.37 ± 3.32	30.70 ± 3.43	23.89 ± 2.91	ND
HpMutS2-E413A	73.77 ± 75.74	^a^	^a^	ND
Holliday junction				
HpMutS2	14.38 ± 0.82	20.77 ± 2.95	22.63 ± 1.391	22.46 ± 1.23
HpMutS2-G338R	21.41 ± 0.93	32.04 ± 4.34	34.02 ± 5.31	ND
HpMutS2-E413A	30.80 ± 1.97	31.72 ± 3.34	46.38 ± 5.60	ND

Dissociation constants were estimated from analysis of Figs. 5b-g. The error values represent standard deviation from at least two different experiments. ND not determined; ^a^, could not be determined

**Fig. 6:**
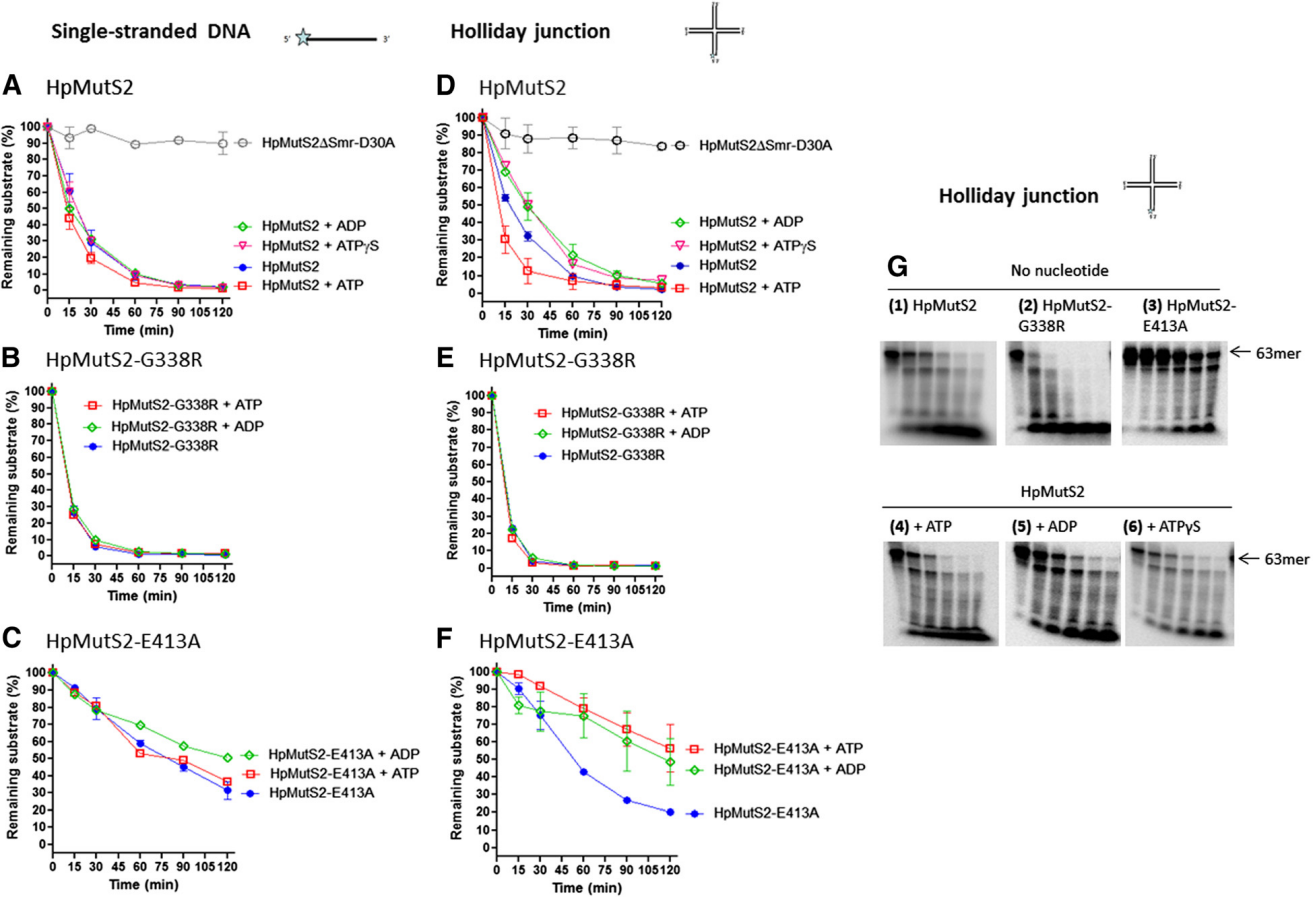
Effect of nucleotide cofactors on nuclease activity of HpMutS2 and mutants. Plots showing time dependent depletion of substrate DNA (**a**, **b**, **c**) Single-stranded DNA and **d**, **e**, **f** Holliday junction. DNA substrates (1 nM) were incubated with HpMutS2 and mutants (150 nM) at 37 °C. Reactions aliquots were removed at 0, 15, 30, 60, 90, and 120 min. The reactions were stopped using (50 mM EDTA + formamide dye) and the products were electrophoresed on urea-PAGE (15 %). The percentage reduction in substrate was calculated by considering DNA without protein as 100 %. Error bars represent standard deviation from two or more different experiments. (**g**, panel 1–6) Time dependent cleavage of Holliday junction by HpMutS2 and mutants. Reactions were performed as described in (A-F). Reactions aliquots were removed at 0, 15, 30, 60, 90, and 120 min

When nuclease activity of wild-type HpMutS2 was monitored in the absence of nucleotides, as expected [7], HpMutS2 degraded both ssDNA (Fig. 6a) and Holliday junction (Figs. 6d and g, panel 1) substrates at comparable rates (Table 3). Interestingly, HpMutS2-G338R exhibited slightly elevated nuclease activities on both DNA substrates (~1.6 fold) (Figs. 6b, e, g, and Table 3). A possible explanation could be inability of nucleotides to fine tune nuclease activity of the protein due to 2.5-fold decreased nucleotide binding efficiencies as well as because of alterations in the tertiary structure of the mutant. On the other hand, HpMutS2-E413A, consistent with its reduced DNA binding affinities (Table 2), exhibited ~4-fold reduced nuclease activities on both DNA substrates (Figs. 6c, f, g, and Table 3). To monitor the effect of nucleotides on the nuclease activity of HpMutS2, nucleotides (1 mM) were added separately to the reaction mixture. In spite of reducing ssDNA binding by the wild-type protein (Table 2), neither ATP nor the other nucleotides tested had significant effects on the ssDNA cleavage ability of the wild-type protein or HpMutS2-G338R and HpMutS2-E413A mutants (Figs. 6a-c). On the other hand, while nucleotides did not alter Holliday junction binding by wild-type HpMutS2 (Table 2) the presence of nucleotides had a significant effect on the Holliday junction cleavage activity (Figs. 6d and g, panel 4–6). While the presence of ATP slightly stimulated the Holliday junction cleavage rates (~1.5-fold), the presence of ADP resulted in slightly reduced rates (~1.5-fold) (Figs. 6d, g, panel 4, 5 and Table 3). Overall, Holliday junction cleavage activity was reduced by ~2.1-fold in the presence of ADP compared to ATP (Fig. 6d and Table 3). The Holliday junction cleavage ability of HpMutS2-G338R was not affected while that of HpMutS2-E413A was further reduced in presence of either ATP or ADP (Figs. 6e and f). ATP hydrolysis assays demonstrated that the Holliday junction stimulates HpMutS2 ATP hydrolysis by ~4-fold (Fig. 1b). A possible interpretation of these results is that ATP hydrolysis up-regulates the nuclease activity of HpMutS2 on Holliday junction. Consistent with this, incubation in the presence of ATPγS, a non-hydrolysable analogue of ATP, resulted in a ~1.5-fold reduced nuclease activity (Fig. 6d, g, panel 6 and Table 3). An inactive nuclease mutant (HpMutS2∆Smr-D30A) [7] taken as a negative control, showed negligible nuclease activities on both the DNA substrates suggesting that the protocol used for proteins preparations was successful in avoiding major contaminating nucleases (Figs. 6a, d and Table 3).

**Table 3:** DNA cleavage rates (pM min^−1^) determined for HpMutS2 and mutants

	No nucleotide	+ ATP	+ ADP	+ ATPγS
Single-stranded DNA				
HpMutS2	24.82 ± 1.15	32.08 ± 5.32	28.11 ± 5.15	28.5 ± 3.68
HpMutS2-G338R	40.19 ± 8.73	40.45 ± 9.52	38.87 ± 8.79	ND
HpMutS2-E413A	6.50 ± 0.55	7.23 ± 0.65	6.90 ± 1.4	ND
HpMutS2ΔSmr-D30A	2.12 ± 1.67	ND	ND	ND
Holliday junction				
HpMutS2	26.49 ± 3.95	37.77 ± 8.59	18.85 ± 1.9	17.44 ± 0.84
HpMutS2-G338R	41.59 ± 9.53	43.7 ± 11.42	41.6 ± 10.27	ND
HpMutS2-E413A	4.92 ± 1.25	1.27 ± 1.01	6.77 ± 3.53	ND
HpMutS2ΔSmr-D30A	3.34 ± 1.67	ND	ND	ND

Reaction rates were estimated by dividing the total product formed by corresponding incubation time obtained from the linear range of enzymatic activity (Figs. 6a-f). The error values represent standard deviation from two or more different time points. ND, not determined

Mutations in the Walker-A and Walker-B motifs of HpMutS2 resulted in minor changes in its DNA binding and nuclease activities. However, these set of observations suggest that HpMutS2 interactions with ATP or ADP may regulate its DNA binding and nuclease activities. Most likely, interaction with ATP or ADP during folding may define a conformational state of the protein. As reported for MutS1 proteins, conformational changes induced by ATP may propagate to other regions of HpMutS2. The nuclease activity of other nucleases is also regulated by ATP binding. Notably, ATP suppresses the non-specific nuclease activity of MutL, a protein recruited by MutS1 during mismatch repair may protect the genomic DNA from degradation [15]. The nuclease activity of Nar71, a nuclease involved in DNA repair is also reduced by ATP [42].

### Intact Walker-A and Walker-B motifs are essential for the anti-recombinase function of HpMutS2

Single point mutations in the conserved Walker-A and Walker-B motifs of HpMutS2 not only disabled the efficiencies of HpMutS2 to bind and hydrolyze ATP respectively but resulted in altered conformation, DNA binding and nuclease activities of the protein. These observations suggest a strong crosstalk between the ATPase, DNA binding and nuclease activities of HpMutS2. Therefore, to study the effect of these mutations *in vivo,* the coding sequences of HpMutS2 or its mutants under the control of the urea promoter were introduced into wild-type and *hpmutS2*-disrupted host strains of *H. pylori*. Western blots performed to verify the expression of proteins confirmed the absence of HpMutS2 in the *hpmutS2*-disrupted strain and showed that the strains into which the different constructs were introduced over-expressed HpMutS2 and its mutants (Additional file 1: Figure S6). Transformation frequencies were determined by monitoring the incorporation of isogenic streptomycin resistant (StrpR) chromosomal DNA. As reported earlier [7] the *hpmutS2*-disrupted strain showed ~8 fold higher recombination frequencies (Table 4). Over-expression of HpMutS2 in the wild-type strain resulted in significantly lower recombination frequencies (Table 4). When HpMutS2-G338R was introduced into the wild-type strain no significant changes were observed in the transformation capacity. On the other hand, over-expression of HpMutS2-E413A in the wild-type strain resulted in ~5-fold higher transformation frequency indicating a dominant negative effect of this mutant.

**Table 4:** Recombination frequencies determined for *H. pylori* strains

Strain genotype	n^a^	Recombinant frequency (x10^−4^)	Relative value	*P* value (MWU)
Wt	30	4.96 (±3.78)	1	
Wt + *hpmutS2*	14	0.963 (±0.97)	0.19	<0.0001
Wt + *hpmutS2-G338R*	15	4.18 (±4.87)	0.84	0.802
Wt + *hpmutS2-E413A*	18	23.4 (±17.8)	4.71	<0.0001
*hpmutS2* ^−^	36	39.2 (±29.2)	7.9	<0.0001
*hpmutS2* ^−^ + *hpmutS2*	27	1.00 (±4.29)	0.20	0.0003
*hpmutS2* ^−^ + *hpmutS2-G338R*	30	25.2 (±22.9)	5.08	<0.0001
*hpmutS2* ^−^ + *hpmutS2-E413A*	21	45.2 (±54.4)	9.11	<0.0001

The recombination frequencies were calculated as the number of streptomycin resistance colonies per recipient cfu. Values correspond to the average and standard deviation. P values were calculated using the Mann–Whitney U test. n^a^ No. of independent determinants

We then analysed the effect of expressing different HpMutS2 proteins in an *hpmutS2*^*−*^ strain of *H. pylori* (Table 4). As expected, over-expression of the wild-type protein not only complemented the hyper-recombination phenotype, but resulted in transformation frequencies significantly lower than that of the wild-type strain, reflecting the effect of the over-expression of a functional protein. Although the HpMutS2-G338R mutant protein exhibited slightly elevated nuclease activities, its expression failed to complement the *hpmutS2*^*−*^ hyper-recombination phenotype. Expression of the HpMutS2-E413A protein in the HpMutS2-null background had no effect on the transformation of the *mutS2-*deficient strain. The inability of the HpMutS2-E413A mutant to complement *hpmutS2*-disruption could be the consequence of its reduced DNA binding and nuclease activities (Tables 2 and 3). Moreover, the dominant negative phenotype we found by its expression in a wild-type strain, suggests that this mutant participates with the wild-type protein in the formation of inactive complexes. Alternatively, the mutant protein could lead to the formation of HpMutS2-E413A:DNA dead end complexes. A similar dominant negative mutator phenotype was observed in case of P-loop mutants of the MSH2 and MSH6 proteins of *Saccharomyces cerevisiae* [19].

## Conclusions

We show here, using various approaches, that ATP and ADP induce conformational changes in HpMutS2. Using mutagenesis, Gly-338 of Walker-A motif and Glu-413 of Walker B motif were shown to be partially responsible for ATP binding and hydrolysis activities of HpMutS2. Biochemical and biophysical characterization of these mutants demonstrated that they show alteration in the conformation, DNA binding and nuclease activities when compared to the wild-type protein. Moreover, it was observed that ATP influences the binding and cleavage of different HR intermediates. Most importantly, we show that both HpMutS2-G338R and HpMutS2-E413A are not able to restore the anti-recombinase function of wild-type HpMutS2 *in vivo* and suggest that both binding and hydrolysis of ATP play important roles in the suppression of recombination by HpMutS2 during tansformation. It is possible that conformational states adopted by HpMutS2 in the presence of ATP or ADP may define its substrate specificity. Moreover, the ATP-ADP exchange may control the anti-recombinase function of HpMutS2 by regulating its DNA binding and nuclease activities. After the import of single-stranded DNA into the cytoplasm, recombination mediator proteins such as DprA and RecA may compete with HpMutS2 to bind ssDNA. DprA has binding affinities close to those of HpMutS2 [43, 44]. In such a case the fate of the transformed DNA depends upon the binding of particular protein. However, the significant reduction of ssDNA binding by HpMutS2 in the presence of ATP may facilitate the binding of DprA and RecA heterodimer to the ssDNA to achieve homologous pairing and strand exchange reactions. HpMutS2 displays ~2-fold lower nuclease activity in presence of ADP on Holliday junction substrates. It is possible that the stimulation of ATP hydrolysis by Holliday junction and D-loop like structures may reduce the inherent nuclease activity of HpMutS2. In such a case, the strand resolution will proceed to favour transformation. In the case of MMR, the capacity of MutS homologues to bind and hydrolyse ATP is essential [17, 19]. Similarly, the highly conserved ATP binding and hydrolysis domains of HpMutS2 play a crucial role in regulation of anti-recombinase activity of the protein. Most importantly we demonstrate that the ATPase, DNA binding and nuclease activities of the HpMutS2, although independent of each other, are essential for its efficient anti-recombinase function.

## Methods

### Construction and purification of HpMutS2 and variants

The cloning of the wild-type *hpmutS2*, *hpmutS2*ΔSmr, and *HpSmr* from *H. pylori* strain 26695 was performed as described in [7]. Briefly the wild-type HpMutS2 was cloned in pET28b expression vector under control of IPTG inducible promoter with C-terminal His_6_ tag. Nucleotide binding Walker-A and hydrolysis Walker-B motifs were identified by multiple sequence alignment using Clustal Omega (http://www.ebi.ac.uk/Tools/msa/clustalo/). The MutS2 sequences from different bacteria were obtained from the NCBI protein database. To generate point mutations in these motifs, QuikChange II Site directed mutagenesis kit (Agilent technologies) was used. Primers and PCR reaction were designed according to the manufacturer’s recommendation. Fallowing pairs of primer were used for mutagenesis (5′-3′):

- HpMutS2-G338R, Forward: GGCGTGAATGCGGGCCGTAAAACCATGCTC,
- Reverse: GAGCATGGTTTTACGGCCCGCATTCACGCC
- HpMutS2-E413A, Forward: CTTTTAGGCGTTGATGCGATCGAGCTAGGGACTGACGC
- Reverse: GCGTCAGTCCCTAGCTCGATCGCATCAACGCCTAAAAGC

A slightly modified version of purification protocol described previously [7] was used to optimize the yield and stability of proteins. *E. coli* Rosetta (DE3) pLysS expression strain transformed with the wild-type HpMutS2 and mutants were grown in 3 litres of LB media containing appropriate antibiotics [kanamycin (25 μg/ml) and chloramphenicol 34 μg/ml]. After induction of proteins with IPTG (0.5 mM) at an optical density of 0.6 at 600 nm the *E. coli* cells were grown overnight at 16 °C. The cells were harvested by centrifugation at 8,000 rpm. The proteins were then purified as described in [7]. Briefly, the His_6_-tagged proteins were first purified by Ni^2+^ chromatography followed by SP-Sepharose ion exchange chromatography. The full length variants of HpMutS2 were purified using Ni^2+^ affinity columns followed by SP-Sepharose ion exchange chromatography. The HpSmr construct was first purified by Ni^2+^ affinity followed by gel filtration chromatography. The homogeneity of the proteins were monitored on SDS-PAGE and the fractions containing pure protein were pooled and dialyzed against storage buffer C (50 mM Tris-Cl pH 8.0, 300 mM NaCl, 5 mM 2-mercaptoethanol and 50 % glycerol) and were stored at −20 °C.

### Preparation of DNA substrates

DNA substrates were prepared as described previously [7]. Briefly, different combinations of chemically synthesized oligonucleotides (Sigma Genosys) (Additional file 1: Tables S1 and S2) were annealed together. The 5′-end radiolabeled substrates were prepared using [γ-^32^P] ATP and T4 polynucleotide kinase (NEB). The free label was removed using Sephadex G-25 spun-column chromatography. 2-fold molar excess of unlabeled complementary oligonucleotides (Additional file 1: Table S2) were added to the labelled DNA in 1X saline-sodium citrate (SSC) buffer (150 mM sodium chloride and 15 mM trisodium citrate adjusted to pH 7.0 with HCl) and were boiled for 10 min at 95 °C. After cooling at room temperature the annealed DNA substrates were electrophoresed on 8 % polyacrylamide gel in 1X TBE buffer. The gel portion containing DNA substrates were identified by autoradiography and were sliced out to elute DNA in 1X TE buffer (10 mM Tris–Cl and 1 mM EDTA). The unlabeled DNA substrates were prepared by heating equimolar concentrations of oligonuclotides (Additional file 1: Tables S1 and S2) at 95 °C for 10 min in 1X SSC buffer followed by cooling at room temperature.

### ATPase assay

ATPase activity of HpMutS2 and mutants was carried out in 10 μl of reaction volume containing 1X buffer A (50 mM Tris pH 8.0, 50 mM NaCl, and 1 mM DTT) containing MgCl_2_ (5 mM), and ATP [γ-^32^P] was used as a tracer. A 1:1000 dilution of 25 μC ATP [γ-^32^P] (3500 Ci/mmol) was used with indicated amount of the cold ATP in all the assays. All reactions were carried out at 37 °C for indicated time and terminated by the addition of EDTA (50 mM). 1 μl aliquot of each reaction mixture was spotted on a polyethylene cellulose TLC sheets. The reaction components [ATP and inorganic phosphate (Pi)] were separated by thin layer chromatography (TLC) using 0.4 M LiCl_2_ and 0.1 mM EDTA as mobile phase. The dried TLC sheets were visualized by phosphorimaging and quantified using Image Gauge (Version 3.0). The kinetic parameters were determined using the Michaelis–Menten equation by non-linear regression of plot of rate of product formation versus substrate concentration using GraphPad Prism 5 software. Reaction velocities were calculated by quantifying the proportion of products formed to un-reacted substrate divided by incubation time. To determine the effect of DNA on ATPase activity 1 μM of DNA substrates were added separately to the reaction mixture.

### Limited proteolysis assays

HpMutS2 (4 μM) was incubated with chymotrypsin (1.25 ng) or in buffer A lacking magnesium at 37 °C for 30 min. The reactions were stopped by addition of protease inhibitor cocktail (Sigma) and the proteolyzed products were separated on SDS-PAGE (10 %). Nucleotide cofactors (1 mM) were pre-incubated with the proteins for 15 min at 37 °C before addition of chymotrypsin. The heat denaturation of proteins was performed by heating them at 95 °C for 10 min. All the reactions were performed in 1X buffer A. Protein bands were detected by staining with Coomassie Brilliant Blue.

### Fluorescence spectroscopic analysis

Fluorescence spectroscopy analysis of HpMutS2 was carried out based on the intrinsic fluorescent signal of tryptophan. The proteins were dialyzed in buffer B (50 mM Tris pH 8.0 and 100 mM NaCl) for 4–6 h with at least two changes of the buffer. Fluorescence intensities were measured on a Shimadzu, RF 5000 spectrofluorimeter using a 1-cm stirred quartz cuvette at 25 °C. The emission spectra were recorded over a wavelength of 300–400 nm with an excitation wavelength of 280 nm. To measure the dissociation constants for ATP and ADP, small aliquots of cofactor were added to proteins (1 μM), and spectra were recorded. Each spectra recorded was an average of three scans. The maximum fluorescence intensities observed at around 340 nm were selected for calculating the difference in intrinsic fluorescence of protein alone and in the presence of ligand. Control titrations were conducted and all the fluorescence emission spectra were corrected by subtracting control spectra. The dissociation constants were calculated graphically using a plot of ΔF/ΔF_max_ versus ligand concentration. ΔF is the magnitude of the difference between the observed fluorescence intensity at a given concentration of ligand and the fluorescence intensity in the absence of ligand and ΔF_max_ is the difference at saturating concentration of ligand. Data were analyzed using GraphPad Prism 5.0 using non-linear regression analysis with single site-specific binding.

### Circular dichroism spectroscopy (CD)

Freshly prepared proteins were dialyzed twice in buffer B for 4–6 h with at least two changes of the buffer. Far-UV CD spectra of proteins (1 μM) were obtained using a Jasco-815 spectropolarimeter equipped with a Peltier stage (Japan Spectroscopic Co., Japan) at 25 °C. The spectra were recorded between 200–300 nm with 20 mdeg sensitivity at a scan speed of 50 nm using 2 mm path length cuvette with a bandwidth of 2 nm. Control titrations were performed and all the CD spectra were corrected by subtracting control spectra. Thermal denaturation of the proteins was monitored at single wavelength (222 nm) by increasing temperature from 25 °C-80 °C. The percentage of unfolded protein was calculated by considering the protein at 25 °C as 100 % folded.

### Electrophoretic mobility shift assay (EMSA)

Radiolabeled DNA substrates (0.16 nM) were incubated with increasing concentrations of HpMutS2 and mutants in buffer A with 1X BSA (0.1 μg/μl). ATP, ADP, and ATPγS (1 mM each) were added separately to the reaction mixture. The NaCl concentration was maintained to 110 mM by adding storage buffer C to the reaction mixture. After incubation of 30 min on ice, the reaction mixtures were electrophoresed on native PAGE (8 %) at 4 °C. The gels were transferred on to Whatman 3 mm paper and dried under vacuum at 80 °C for 45 min. The gels were visualized by phosphorimaging and quantified using Image Gauge (Version 3.0). The dissociation constants were determined by non-linear regression analysis and calculated by plotting the percentage of bound DNA versus concentration of protein using “One site - Specific binding with Hill slope” equation of the GraphPad Prism 5 software.

### Nuclease assays

Radiolabeled DNA substrates were incubated with HpMutS2 and mutants in buffer D (50 mM Tris pH 8.0, 50 mM NaCl, 1 mM DTT, and 5 mM MgCl_2_). ATP, ADP, and ATPγS (1 mM each) were added separately to the reaction mixtures. 20 μl reaction aliquots were reomoved at 0, 15, 30, 60, 90, and 120 min. Reactions were stopped by adding EDTA (50 mM) + formammide dye (98 % formamide, 0.1 % bromophenol blue and 0.1 % xylene cyanol). The DNA substrates were denatured at 95 °C for 15 min and the reaction mixtures were separated on denaturing polyacrylamide gel (15 %) containing urea (7 M). The results were visualized and analysed as described earlier. DNA cleavage rates were estimated by dividing the total product formed (pM) in linear range of enzymatic activity by corresponding incubation time (min).

### Gel filtration chromatography

Superose-6 HR 10/30 column (GE Healthcare) was used to determine the elution volumes of proteins. The column was equilibrated with buffer B. The flow rate was maintained at 0.3 ml/min and the elution profile was monitored by the absorbance at 280 nm. The void volume (*V*_o_) was determined using Blue Dextran (2000 kDa). The standard molecular mass markers were obtained from BioRad [Thyroglobulin (670 kDa), γ-globulin (158 kDa), ovalbumin (44 kDa), myoglobin (17 kDa), vitamin B12 (1.35 kDa)]. The molecular mass and oligomerization status of proteins were determined from the plot of *V*_e_/*V*_o_ versus log of molecular mass.

### Construction of *H. pylori* strains and recombination assay

The construction of *hpmutS2-*disrupted strain, complements, and calculations of recombination frequencies were performed as described in [7]. Briefly, *H. pylori* strain 26695 was used to disrupt *hpmutS2* gene by allelic replacement using a chloramphenicol resistance cassette and to generate integrations. The wild-type and mutants of HpMutS2 preceded by *ure*A promoter and containing an apramycin resistance cassette were integrated into a non-essential *ure*A locus. The correct constructions were verified by PCR and the expression of proteins was monitored by Western blots. Recombination frequencies were determined by rate of incorporation of streptomycin resistant isogenic total chromosomal DNA (200 ng) per cfu. P values were calculated using the Mann–Whitney U test. At least two independent clones of each construction were used to determine the recombination frequencies. All DNA constructs were introduced using natural transformation. *H. pylori* was grown on BAB plates at 37 °C under microaerophillic conditions.

## Availability of supporting data

The data supporting the results of this article are included within Additional file 1: Figures S1-S6 and Additional file 1: Tables S1-S2.

## Additional file

Additional files 1: Figure S1. Multiple sequence alignment of MutS2 proteins from different *H. pylori* strains. Multiple sequence alignment of MutS2 proteins from different bacteria was carried out using Clustal Omega (http://www.ebi.ac.uk/Tools/msa/clustalo/). The protein sequences were obtained from NCBI protein database. The conserved nucleotide binding Walker-A motif and nucleotde hydrolysis Walker-B motifs are framed. The arrow indicates *H. pylori* MutS2 protein sequence. **Figure S2.** ATPase activity of HpMutS2 and variants. (A) Divalent metal ion requirement for ATPase activity of HpMutS2. Mentioned divalent metal ions (5 mM each) were incubated separately with cold ATP (100 μM) with (+) or without (−) HpMutS2 (45 nM). After incubation at 37 °C for 30 min the reactions were stopped by EDTA (50 mM) and the products were separated by TLC. ATP [γ-^32^P] was used as tracer to monitor the product formation. (B) Time dependent hydrolysis of ATP by HpMutS2. A mixture of cold ATP (1 mM) and ATP [γ-^32^P] was incubated in presence of HpMutS2 (45 nM) at 37 °C . The reaction aliquots were removed at indicated time points. The reactions were stopped by adding EDTA (50 mM) and products were separated by TLC. Product formed was calculated by quantifying the proportion of products formed to un-reacted substrate. (C) Effect of HpSmr domain deletion on ATPase activity of HpMutS2. Increasing concentrations of proteins were incubated with a mixture of cold ATP (1 mM) and ATP [γ-^32^P] at 37 °C for 30 min. The reactions were processed as described in (A). Product formed was calculated by quantifying the proportion of products formed to un-reacted substrate. **Figure S3.** Limited proteolysis. Proteins (4 μM) were incubated with chymotrypsin (1.25 ng) for 30 min at 37 °C. The heat denaturation of proteins was performed by heating them at 95 °C for 10 min. All the reactions were performed in 1X buffer A (50 mM Tris pH 8.0, 50 mM NaCl, and 1 mM DTT). Reactions were stopped by adding 1X protease inhibitor cocktail (Sigma), heat denatured and the products were separated on SDS-PAGE (10 %). Lane M: Molecular weight marker. Protein bands were detected by staining with Coomassie Brilliant Blue. Lane M: Molecular weight marker. **Figure S4.** (A-E) Effect of nucleotides on DNA binding properties of HpMutS2. Electrophoretic mobility shift assays were performed with indicated DNA substrates (0.16 nM) without and with nucleotides (1 mM). After incubation for 30 min on ice, the reaction mixture was electrophoresed on polyacrylamide gel (8 %). Schematic representation of DNA substrates used are shown at the left side of autoradiographs. **Figure S5.** Chase experiments. A preformed ssDNA-HpMutS2 complex was chased with increasing concentrations of (A) ATP (0.25–5 mM) and (B) ssDNA (7.5–500 nM). (C) A preformed SC pUC19 DNA-HpMutS2 complex was chased with increasing concentrations of ATP (0.25–5 mM) . The reactions were performed at 37 °C and the reaction mixture was electrophoresed on PAGE (8 %) in case of (A and B) or an agarose gel (0.8 %) in case of (C). The amount of displaced DNA was estimated by assuming the DNA without protein as 100 %. **Figure S6.** Western blot. Equal numbers of exponentially growing *H. pylori* cells were lysed in Laemmli buffer (1X) by boiling at 95 °C for 5 min. The proteins were separated by SDS-PAGE (10 %). The HpMutS2 and mutants were detected by probing with polyclonal antibodies against HpMutS2. **Table S1.** Sequence of oligonucleotides used to prepare DNA substrates. **Table S2.** DNA substrates used in this study (* represents position of ^32^P). (PDF 636 kb)

## Abbreviations

CD: circular dichroism
HR: homologous recombination
NT: natural transformation
Smr: small MutS related

## References

[1] Israel DA, Salama N, Arnold CN, Moss SF, Ando T, Wirth HP (2001). *Helicobacter pylori* strain-specific differences in genetic content, identified by microarray, influence host inflammatory responses. J Clin Invest.

[2] Dorer MS, Cohen IE, Sessler TH, Fero J, Salama NR (2013). Natural competence promotes *Helicobacter pylori* chronic infection. Infect Immun.

[3] Kang J, Blaser MJ (2006). Bacterial populations as perfect gases: genomic integrity and diversification tensions in *Helicobacter pylori*. Nat Rev Microbiol.

[4] Dorer MS, Sessler TH, Salama NR (2011). Recombination and DNA repair in *Helicobacter pylori*. Annu Rev Microbiol.

[5] Pinto AV, Mathieu A, Marsin S, Veaute X, Ielpi L, Labigne A (2005). Suppression of homologous and homeologous recombination by the bacterial MutS2 protein. Mol Cell.

[6] Fukui K, Nakagawa N, Kitamura Y, Nishida Y, Masui R, Kuramitsu S (2008). Crystal structure of MutS2 endonuclease domain and the mechanism of homologous recombination suppression. J Biol Chem.

[7] Damke PP, Dhanaraju R, Marsin S, Radicella JP, Rao DN (2015). The nuclease activities of both the Smr domain and an additional LDLK motif are required for an efficient anti-recombination function of *Helicobacter pylori* MutS2. Mol Microbiol.

[8] Kang J, Huang S, Blaser MJ (2005). Structural and functional divergence of MutS2 from bacterial MutS1 and eukaryotic MSH4-MSH5 homologs. J Bacteriol.

[9] Lin Z, Nei M, Ma H (2007). The origins and early evolution of DNA mismatch repair genes--multiple horizontal gene transfers and co-evolution. Nucleic Acids Res.

[10] Sachadyn P (2010). Conservation and diversity of MutS proteins. Mutat Res.

[11] Biswas I, Vijayvargia R (2000). Heteroduplex DNA and ATP induced conformational changes of a MutS mismatch repair protein from *Thermus aquaticus*. Biochem J.

[12] Joshi A, Sen S, Rao BJ (2000). ATP-hydrolysis-dependent conformational switch modulates the stability of MutS-mismatch complexes. Nucleic Acids Res.

[13] Lamers MH, Georgijevic D, Lebbink JH, Winterwerp HH, Agianian B, de Wind N (2004). ATP increases the affinity between MutS ATPase domains. Implications for ATP hydrolysis and conformational changes. J Biol Chem.

[14] Qiu R, DeRocco VC, Harris C, Sharma A, Hingorani MM, Erie DA (2012). Large conformational changes in MutS during DNA scanning, mismatch recognition and repair signalling. Embo J.

[15] Shimada A, Kawasoe Y, Hata Y, Takahashi TS, Masui R, Kuramitsu S (2013). MutS stimulates the endonuclease activity of MutL in an ATP-hydrolysis-dependent manner. Febs J.

[16] Antony E, Hingorani MM (2003). Mismatch recognition-coupled stabilization of Msh2-Msh6 in an ATP-bound state at the initiation of DNA repair. Biochemistry.

[17] Geng H, Sakato M, DeRocco V, Yamane K, Du C, Erie DA (2012). Biochemical analysis of the human mismatch repair proteins hMutSalpha MSH2(G674A)-MSH6 and MSH2-MSH6(T1219D). J Biol Chem.

[18] Heinen CD, Cyr JL, Cook C, Punja N, Sakato M, Forties RA (2011). Human MSH2 (hMSH2) protein controls ATP processing by hMSH2-hMSH6. J Biol Chem.

[19] Studamire B, Quach T, Alani E (1998). *Saccharomyces cerevisiae* Msh2p and Msh6p ATPase activities are both required during mismatch repair. Mol Cell Biol.

[20] Wilson T, Guerrette S, Fishel R (1999). Dissociation of mismatch recognition and ATPase activity by hMSH2-hMSH3. J Biol Chem.

[21] Mendillo ML, Putnam CD, Mo AO, Jamison JW, Li S, Woods VL (2010). Probing DNA- and ATP-mediated conformational changes in the MutS family of mispair recognition proteins using deuterium exchange mass spectrometry. J Biol Chem.

[22] Junop MS, Obmolova G, Rausch K, Hsieh P, Yang W (2001). Composite active site of an ABC ATPase: MutS uses ATP to verify mismatch recognition and authorize DNA repair. Mol Cell.

[23] Fukui K, Kosaka H, Kuramitsu S, Masui R (2007). Nuclease activity of the MutS homologue MutS2 from *Thermus thermophilus* is confined to the Smr domain. Nucleic Acids Res.

[24] Jeong E, Jo H, Kim TG, Ban C (2012). Characterization of multi-functional properties and conformational analysis of MutS2 from *Thermotoga maritima* MSB8. PLoS One.

[25] Gradia S, Acharya S, Fishel R (1997). The human mismatch recognition complex hMSH2-hMSH6 functions as a novel molecular switch. Cell.

[26] Bjornson KP, Allen DJ, Modrich P (2000). Modulation of MutS ATP hydrolysis by DNA cofactors. Biochemistry.

[27] Hollingsworth NM, Ponte L, Halsey C (1995). MSH5, a novel MutS homolog, facilitates meiotic reciprocal recombination between homologs in *Saccharomyces cerevisiae* but not mismatch repair. Genes Dev.

[28] Snowden T, Acharya S, Butz C, Berardini M, Fishel R (2004). hMSH4-hMSH5 recognizes Holliday Junctions and forms a meiosis-specific sliding clamp that embraces homologous chromosomes. Mol Cell.

[29] Antony E, Hingorani MM (2004). Asymmetric ATP binding and hydrolysis activity of the *Thermus aquaticus* MutS dimer is key to modulation of its interactions with mismatched DNA. Biochemistry.

[30] Mazur DJ, Mendillo ML, Kolodner RD (2006). Inhibition of Msh6 ATPase activity by mispaired DNA induces a Msh2(ATP)-Msh6(ATP) state capable of hydrolysis-independent movement along DNA. Mol Cell.

[31] Iaccarino I, Marra G, Dufner P, Jiricny J (2000). Mutation in the magnesium binding site of hMSH6 disables the hMutSalpha sliding clamp from translocating along DNA. J Biol Chem.

[32] Obmolova G, Ban C, Hsieh P, Yang W (2000). Crystal structures of mismatch repair protein MutS and its complex with a substrate DNA. Nature.

[33] Li GM (2008). Mechanisms and functions of DNA mismatch repair. Cell Res.

[34] Blackwell LJ, Martik D, Bjornson KP, Bjornson ES, Modrich P (1998). Nucleotide-promoted release of hMutSalpha from heteroduplex DNA is consistent with an ATP-dependent translocation mechanism. J Biol Chem.

[35] Kunkel TA, Erie DA (2005). DNA mismatch repair. Annu Rev Biochem.

[36] Mortier-Barriere I, Velten M, Dupaigne P, Mirouze N, Pietrement O, McGovern S (2007). A key presynaptic role in transformation for a widespread bacterial protein: DprA conveys incoming ssDNA to RecA. Cell.

[37] Orillard E, Radicella JP, Marsin S (2011). Biochemical and cellular characterization of *Helicobacter pylori* RecA, a protein with high-level constitutive expression. J Bacteriol.

[38] Lebbink JH, Fish A, Reumer A, Natrajan G, Winterwerp HH, Sixma TK (2010). Magnesium coordination controls the molecular switch function of DNA mismatch repair protein MutS. J Biol Chem.

[39] Gradia S, Subramanian D, Wilson T, Acharya S, Makhov A, Griffith J (1999). hMSH2-hMSH6 forms a hydrolysis-independent sliding clamp on mismatched DNA. Mol Cell.

[40] Jacobs-Palmer E, Hingorani MM (2007). The effects of nucleotides on MutS-DNA binding kinetics clarify the role of MutS ATPase activity in mismatch repair. J Mol Biol.

[41] Selmane T, Schofield MJ, Nayak S, Du C, Hsieh P (2003). Formation of a DNA mismatch repair complex mediated by ATP. J Mol Biol.

[42] Guy CP, Majernik AI, Chong JP, Bolt EL (2004). A novel nuclease-ATPase (Nar71) from archaea is part of a proposed thermophilic DNA repair system. Nucleic Acids Res.

[43] Dwivedi GR, Sharma E, Rao DN (2013). *Helicobacter pylori* DprA alleviates restriction barrier for incoming DNA. Nucleic Acids Res.

[44] Wang W, Ding J, Zhang Y, Hu Y, Wang DC (2014). Structural insights into the unique single-stranded DNA-binding mode of *Helicobacter pylori* DprA. Nucleic Acids Res.

