# Correction to: Mutations in the nucleotide binding and hydrolysis domains of helicobacter pylori MutS2 lead to altered biochemical activities and inactivation of its in vivo function

**DOI:** 10.1186/s12866-019-1567-7

**Published:** 2019-08-19

**Authors:** Prashant P. Damke, Rajkumar Dhanaraju, Stéphanie Marsin, J. Pablo Radicella, Desirazu N. Rao

**Affiliations:** 1 Department of Biochemistry, Indian Institute of Science, Bangalore, 560012 India; 2 CEA, Institute of Cellular and Molecular Radiobiology, Fontenay aux Roses, France; 3 INSERM UMR967, Fontenay aux Roses, France; 4 Universités Paris Diderot et Paris Sud, Fontenay aux Roses, France

**Correction to: BMC Microbiol**

**https://doi.org/10.1186/s12866-016-0629-3**

Following publication of the original article [1], the authors notified us of an error in the presentation of Fig. 6G.

**Fig. 6:**
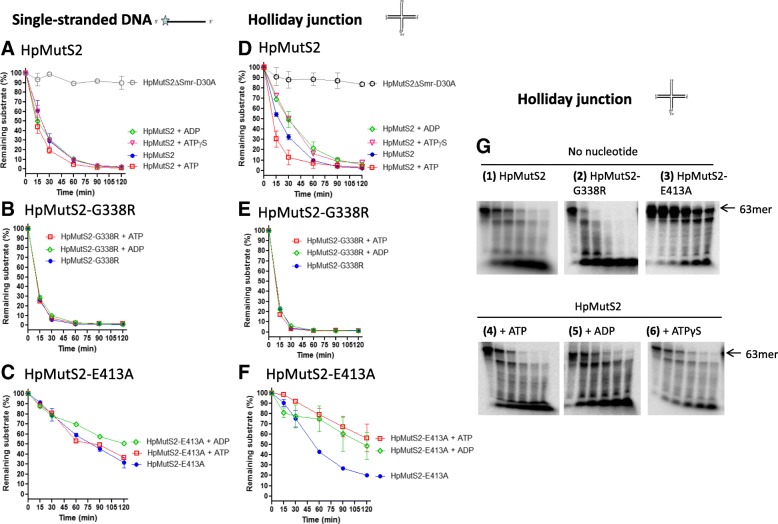
Effect of nucleotide cofactors on nuclease activity of HpMutS2 and mutants. Plots showing time dependent depletion of substrate DNA (**a**, **b**, **c**) Single-stranded DNA and **d**, **e**, **f** Holliday junction. DNA substrates (1 nM) were incubated with HpMutS2 and mutants (150 nM) at 37 °C. Reactions aliquots were removed at 0, 15, 30, 60, 90, and 120 min. The reactions were stopped using (50 mM EDTA + formamide dye) and the products were electrophoresed on urea-PAGE (15 %). The percentage reduction in substrate was calculated by considering DNA without protein as 100 %. Error bars represent standard deviation from two or more different experiments. (**g**, panel 1–6) Time dependent cleavage of Holliday junction by HpMutS2 and mutants. Reactions were performed as described in (A-F). Reactions aliquots were removed at 0, 15, 30, 60, 90, and 120 min

In the published version panels 5 and 6 of the Fig. 6G are similar, but the nuclease assays contained two different nucleotide cofactors. During assembly and handling of the images for this figure, the same image, albeit with different contrast, was used unintentionally for panels 5 and 6. This image corresponded to panel 6.

The assay addressed the effect of ADP (Fig. 6G, panel 5) and ATPγS (Fig. 6G, panel 6) on the nuclease activity of HpMutS2 on Holliday junction substrate. In absence of cofactors (Fig. 6D, and 6G panel 1), HpMutS2 degrades Holliday junction at 26.49 ± 3.95 pM min-1 (Table 3). The addition of ADP and ATPγS reduced the nuclease activity of HpMutS2 by ~ 1.5-fold, resulting in the cleavage rates of 18.85 ± 1.9 pM min-1 and 17.44 ± 0.84 pM min-1, respectively.

The quantification of the assays shown in Fig. 6D were derived from the correct gel images and therefore Fig. 6D and DNA cleavage rates presented in Table 3 remains unchanged.

Corrected Figure ^6 is presented below:

## References

[1] Damke et al. (2016) Mutations in the nucleotide binding and hydrolysis domains of Helicobacter pylori MutS2 lead to altered biochemical activities and inactivation of its in vivo function (2016) 16:14 DOI: 10.1186/s12866-016-0629-3. 10.1186/s12866-016-0629-3 PMC4739419 26843368

