# Supplementary material for: Mutations in the nucleotide binding and hydrolysis domains of Helicobacter pylori MutS2 lead to altered biochemical activities and inactivation of its in vivo function
Source: BMC Microbiol. 2016 Feb 3;16:14. doi: 10.1186/s12866-016-0629-3 (PMC4739419; doi:10.1186/s12866-016-0629-3)
Supplement: Additional files 1: Figure S1. — Multiple sequence alignment of MutS2 proteins from different H. pylori strains. Multiple sequence alignment of MutS2 proteins from different bacteria was carried out using Clustal Omega (http://www.ebi.ac.uk/Tools/msa/clustalo/). The protein sequences were obtained from NCBI protein database. The conserved nucleotide binding Walker-A motif and nucleotde hydrolysis Walker-B motifs are framed. The arrow indicates H. pylori MutS2 protein sequence. Figure S2. ATPase activity of HpMutS2 and variants. (A) Divalent metal ion requirement for ATPase activity of HpMutS2. Mentioned divalent metal ions (5 mM each) were incubated separately with cold ATP (100 μM) with (+) or without (−) HpMutS2 (45 nM). After incubation at 37 °C for 30 min the reactions were stopped by EDTA (50 mM) and the products were separated by TLC. ATP [γ-32P] was used as tracer to monitor the product formation. (B) Time dependent hydrolysis of ATP by HpMutS2. A mixture of cold ATP (1 mM) and ATP [γ-32P] was incubated in presence of HpMutS2 (45 nM) at 37 °C . The reaction aliquots were removed at indicated time points. The reactions were stopped by adding EDTA (50 mM) and products were separated by TLC. Product formed was calculated by quantifying the proportion of products formed to un-reacted substrate. (C) Effect of HpSmr domain deletion on ATPase activity of HpMutS2. Increasing concentrations of proteins were incubated with a mixture of cold ATP (1 mM) and ATP [γ-32P] at 37 °C for 30 min. The reactions were processed as described in (A). Product formed was calculated by quantifying the proportion of products formed to un-reacted substrate. Figure S3. Limited proteolysis. Proteins (4 μM) were incubated with chymotrypsin (1.25 ng) for 30 min at 37 °C. The heat denaturation of proteins was performed by heating them at 95 °C for 10 min. All the reactions were performed in 1X buffer A (50 mM Tris pH 8.0, 50 mM NaCl, and 1 mM DTT). Reactions were stopped by adding 1X protease inhibitor cocktail (Sigma), heat [file 12866_2016_629_MOESM1_ESM.pdf]

Supporting data

Mutations in the nucleotide binding and hydrolysis domains of *Helicobacter pylori* MutS2 lead to altered biochemical activities and inactivation of its in vivo function

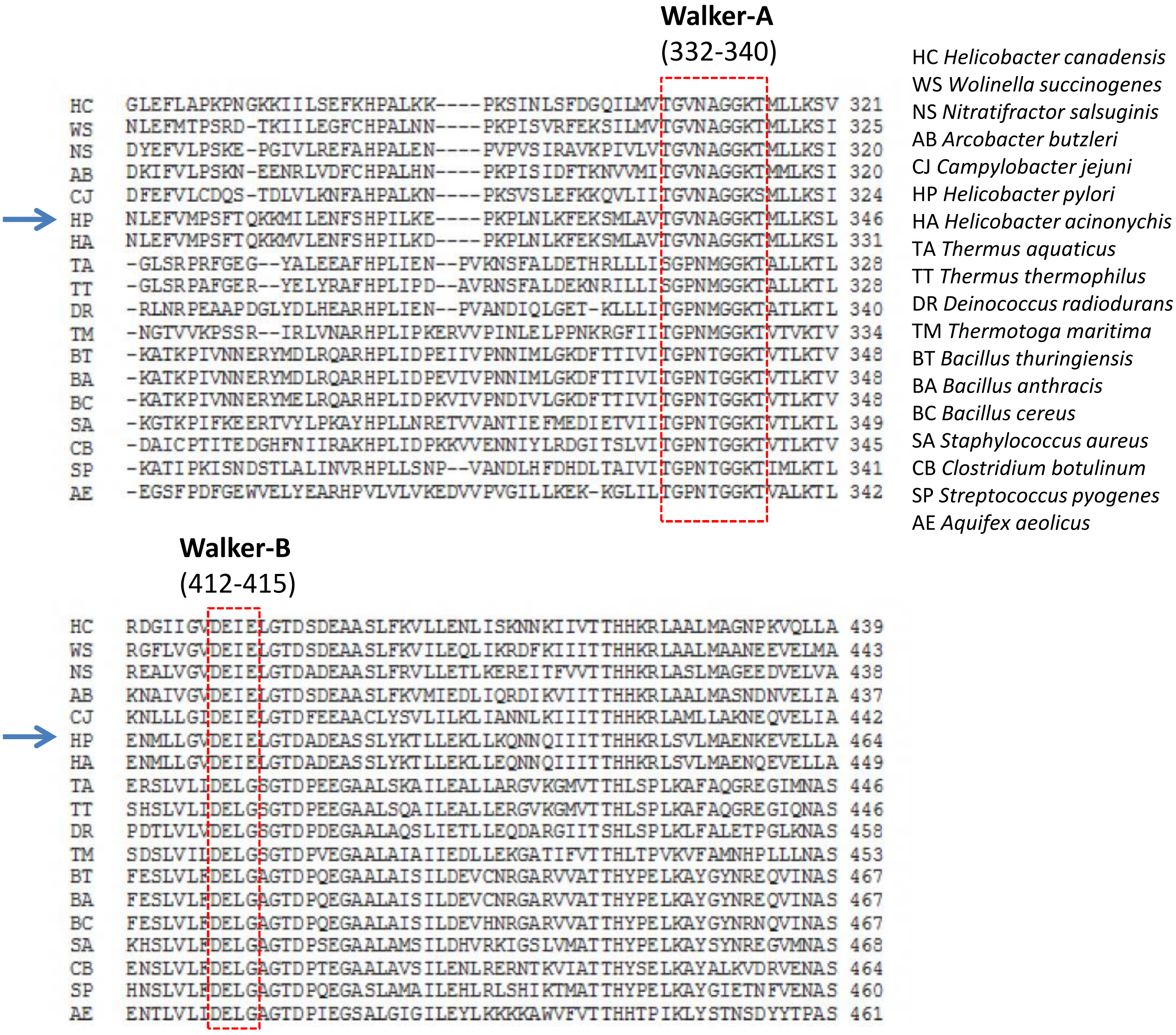

Figure S1. Multiple sequence alignment of MutS2 proteins from different bacteria. Multiple sequence alignment of MutS2 proteins from different bacteria was carried out using ClustalW2 (http://www.ebi.ac.uk/Tools/msa/clustalw2/). The protein sequences were obtained from NCBI protein database. The conserved nucleotide binding Walker-A motif and nucleotde hydrolysis Walker-B motifs are framed. The arrow indicates *H. pylori* MutS2 protein sequence.

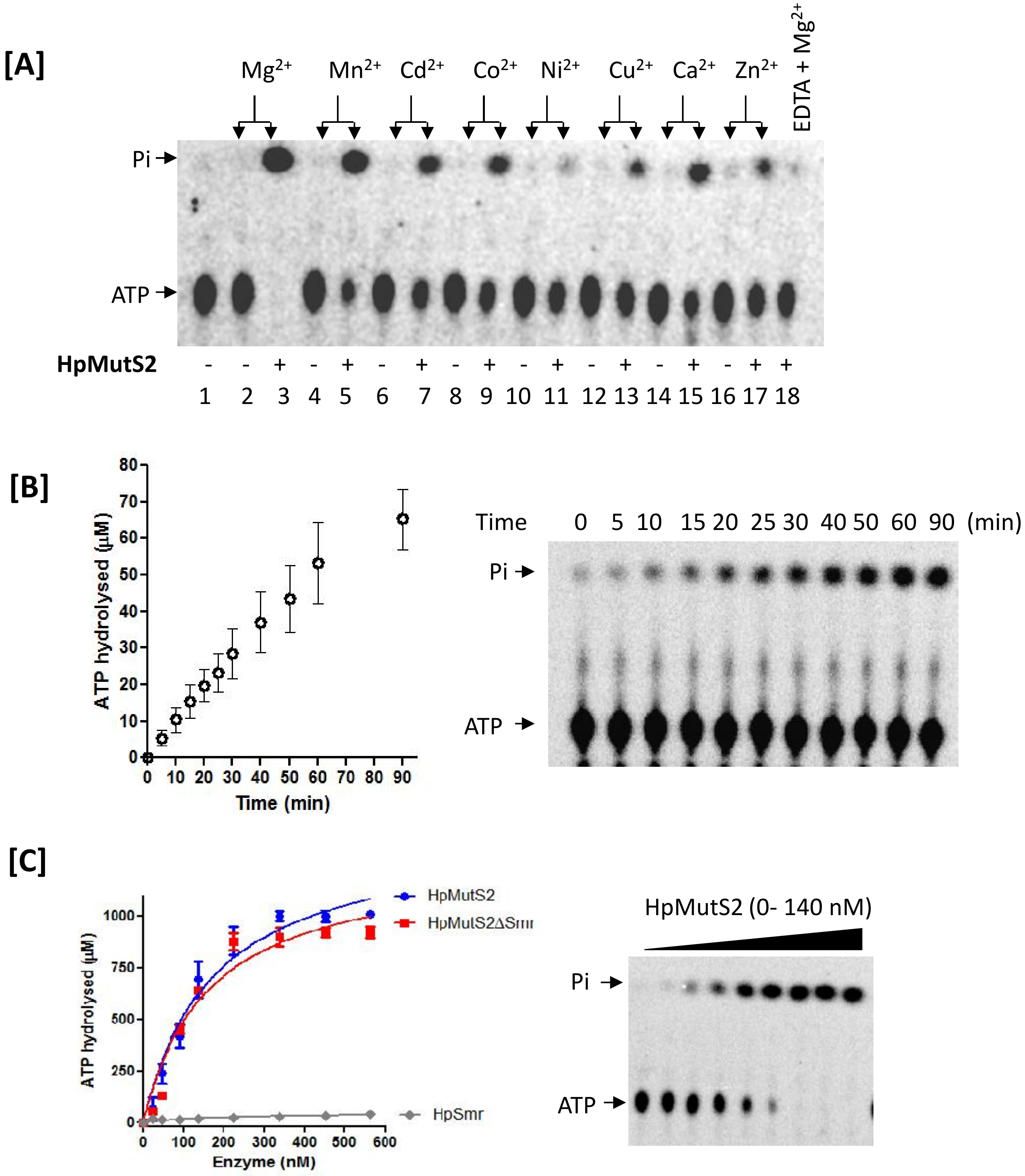

Figure S2. ATPase activity of HpMutS2 and variants. (A) Divalent metal ion requirement for ATPase activity of HpMutS2. Mentioned divalent metal ions (5 mM each) were incubated separately with cold ATP (100 µM) with (+) or without (-) HpMutS2 (45 nM). After incubation at 37 ^{o}C for 30 min the reactions were stopped by EDTA (50 mM) and the products were separated by TLC. ATP [γ-^{32}P] was used as tracer to monitor the product formation. **(B)** Time dependent hydrolysis of ATP by HpMutS2. A mixture of cold ATP (1 mM) and ATP [γ-^{32}P] was incubated in presence of HpMutS2 (45 nM) at 37 ^{0}C . The reaction aliquots were removed at indicated time points. The reactions were stopped by adding EDTA (50 mM) and products were separated by TLC. Product formed was calculated by quantifying the proportion of products formed to un-reacted substrate. **(C) Effect of HpSmr domain deletion on ATPase activity of HpMutS2.** Increasing concentrations of proteins were incubated with a mixture of cold ATP (1 mM) and ATP [γ-^{32}P] at 37 ^{0}C for 30 min. The reactions were processed as described in (A). Product formed was calculated by quantifying the proportion of products formed to un-reacted substrate.

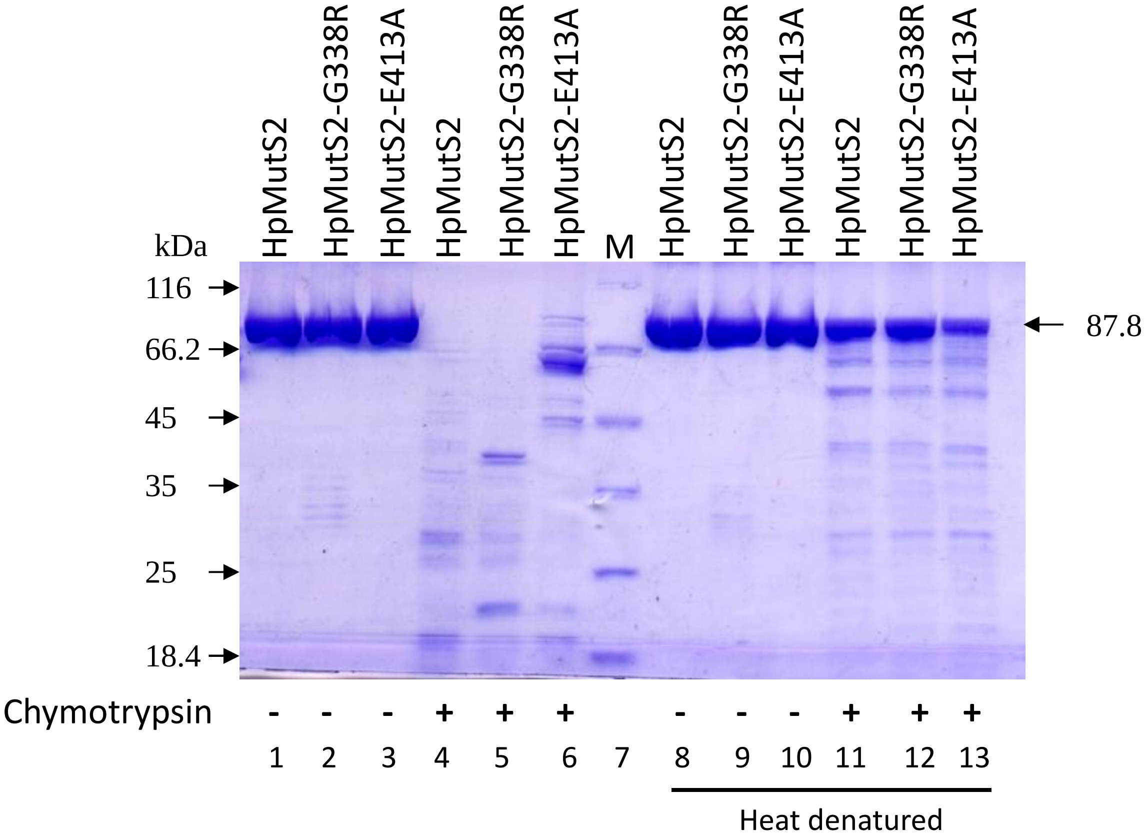

Figure S3. **Limited proteolysis**. Proteins (4 µM) were incubated with chymotrypsin (1.25 ng) for 30 min at 37 ^{o}C. The heat denaturation of proteins was performed by heating them at 95 ^{0}C for 10 min. All the reactions were performed in 1X buffer A (50 mM Tris pH 8.0, 50 mM NaCl, and 1 mM DTT). Reactions were stopped by adding 1X protease inhibitor cocktail (Sigma), heat denatured and the products were separated on SDS-PAGE (10 %). Lane M: Molecular weight marker. Protein bands were detected by staining with Coomassie Brilliant Blue. Lane M: Molecular weight marker

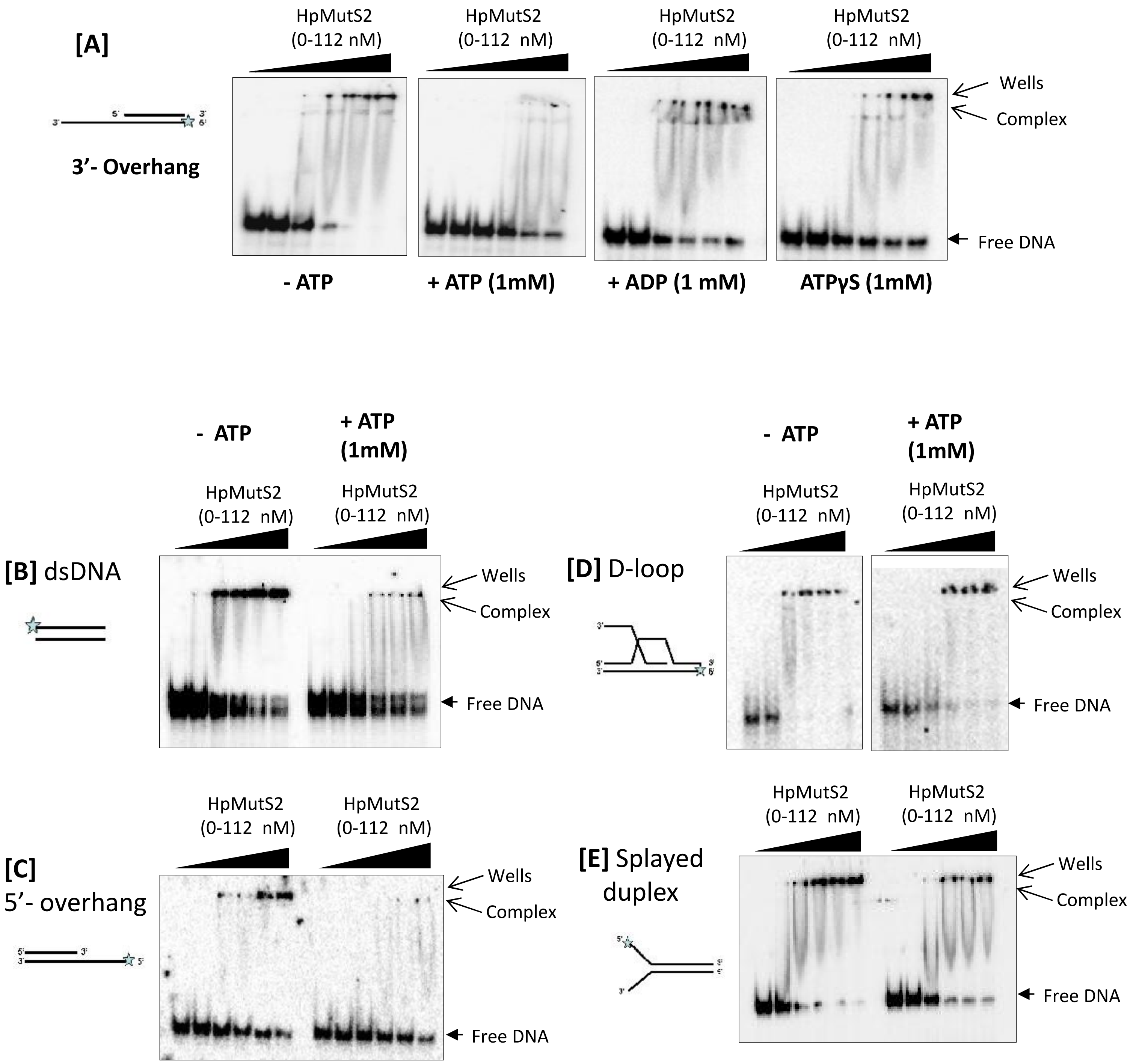

Figure S4. **(A-E) Effect of nucleotides on DNA binding properties of HpMutS2.** Electrophoretic mobility shift assays were performed with indicated DNA substrates (0.16 nM) without and with nucleotides (1 mM). After incubation for 30 min on ice, the reaction mixture was electrophoresed on polyacrylamide gel (8 %). Schematic representation of DNA substrates used are shown at the left side of autoradiographs.

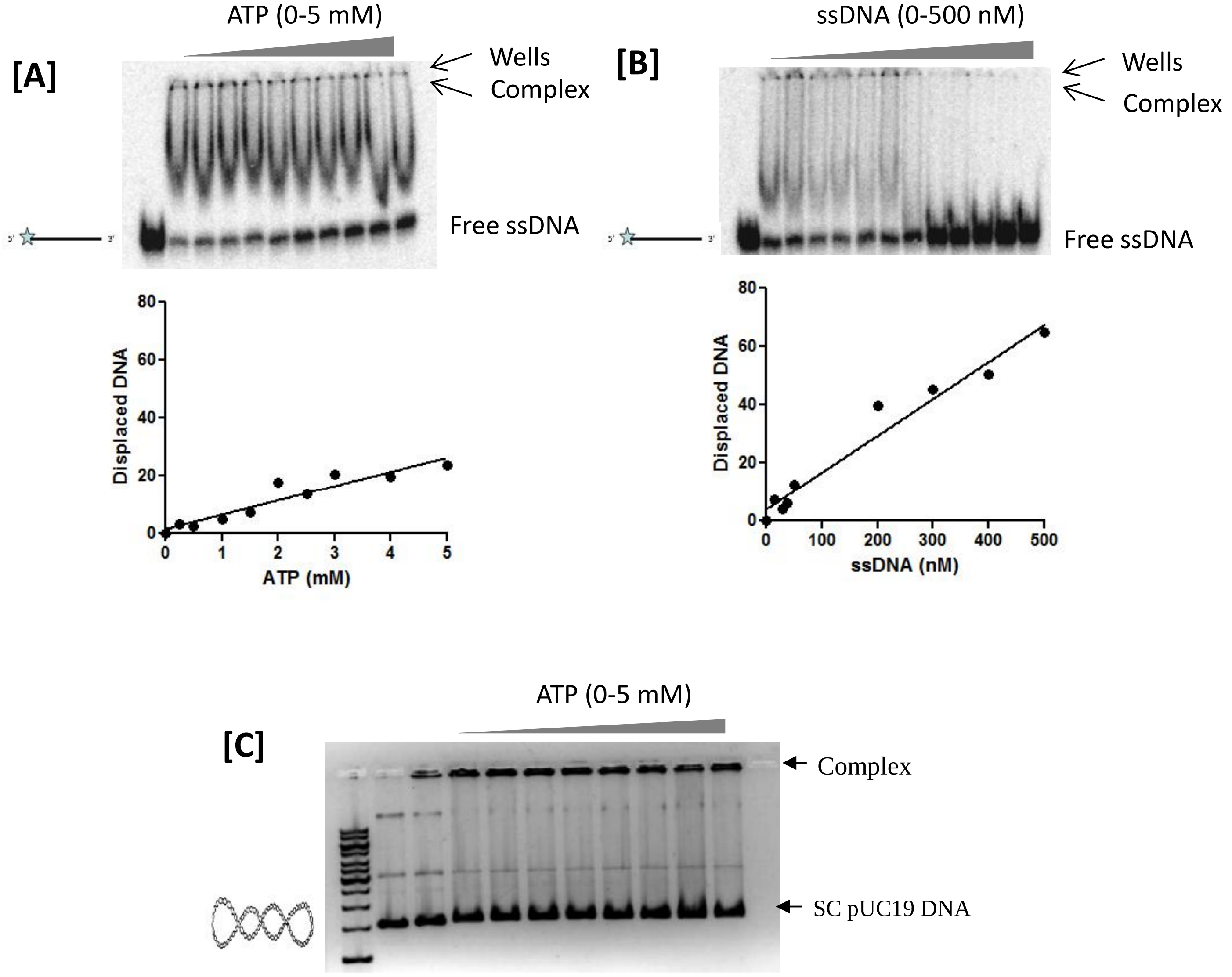

Figure S5. **Chase experiments**. A preformed ssDNA-HpMutS2 complex was chased with increasing concentrations of **(A)** ATP (0.25-5 mM) and **(B)** ssDNA (7.5-500 nM). **(C)** A preformed SC pUC19 DNA-HpMutS2 complex was chased with increasing concentrations of ATP (0.25-5 mM) . The reactions were performed at 37 ^{o}C and the reaction mixture was electrophoresed on PAGE (8 %) in case of (A and B) or an agarose gel (0.8 %) in case of (C). The amount of displaced DNA was estimated by assuming the DNA without protein as 100 %.

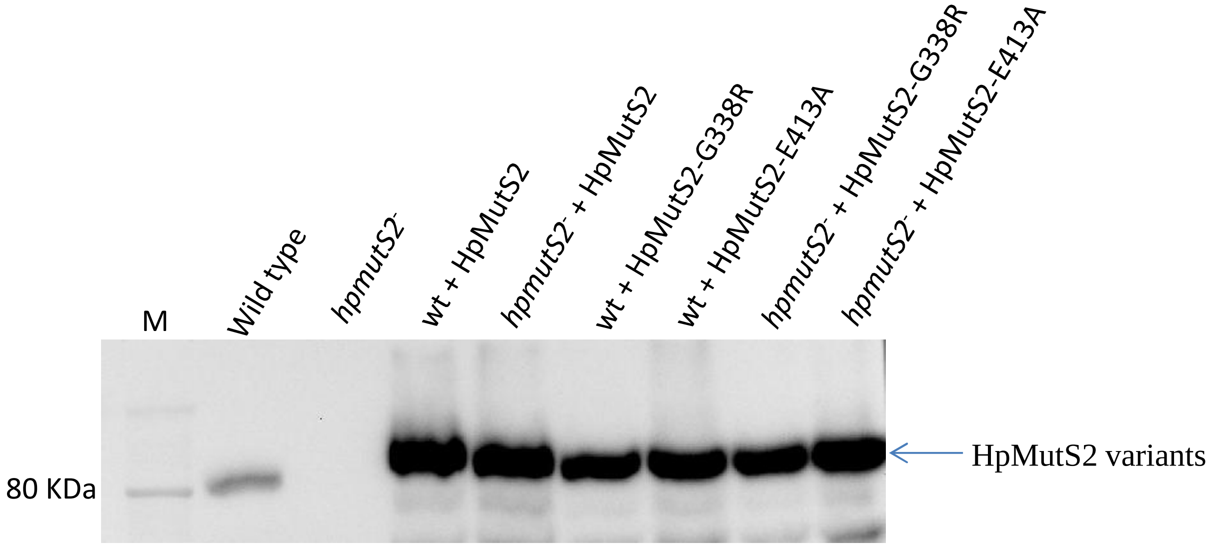

Figure S6. Western blot. Equal numbers of exponentially growing *H. pylori* cells were lysed in Laemmli buffer (1X) by boiling at 95 ^{o}C for 5 min. The proteins were separated by SDS-PAGE (10 %). The HpMutS2 and mutants were detected by probing with polyclonal antibodies against HpMutS2.

Table S1. Sequence of oligonucleotides used to prepare DNA substrates

Description	Size in bases	Sequence (5'-3')
ODN 0	63	GACGCTGCCGAATTCTACCAGTGCCTTGCTACATGGAGCTGTCTGGAGGATCC GACTATCGAT
ODN1	61	GACGCTGCCGAATTCTACCAGTGCCTTGCTAGGACATCTTTGCCCACCTGCAG GTTCACCC
ODN2	28	TGGGTGAACCTGCAGGTGGGCAAAGATG
ODN3	28	GGAGCTGTCTGGAGGATCCGACTATCGA
ODN4	63	ATCGATAGTCGGATCCTCCAGACAGCTCCATGTAGCAAGGCACTGGTAGAATTC GGCAGCGTC
ODN7	28	GACGCTGCCGAATTCTACCAGTGCCTTG
ODN 10	61	GGGTGAACCTGCAGGTGGGCAAAGATGTCCATTAGTGGATCCTTAGCACCGTT GTAAGACG
ODN 11	63	CGTCTTACAACGGTGCTAAGGATGCACTAATCATGGAGCTGTCTAGAGGATCC GACTATCGAT
DL	60	CCGCTACCAGTGATCACCAATGGATTGCTAGGACATCTTTGCCCACCTGCAGGT TCACCC
DL-forward	50	GGGTGAACCTGCAGGTGATGTTAGCCATGACCTGTGATCACTGGTAGCGG
DL-reverse	33	TAAGACTGGACTCAGCTAGGTCATGGCTAACAT

Table S2. DNA substrates used in this study (* represents position of ^{32}P)

DNA Substrate	Description	Composition
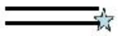 Duplex DNA	63 base pair duplex	ODN 0 + ODN 4
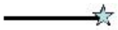 Single-stranded DNA	63 mer ssDNA	ODN 4
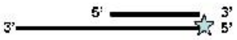 3'-Overhang	27 base pair duplex with 34 mer 3' overhang	ODN 3 + ODN 4
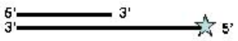 5'-Overhang	28 base pair duplex with 35mer 5' overhang	ODN 4 + ODN 7
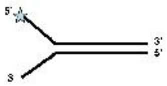 Splayed-duplex	31 base pair duplex with 30mer overhang at 3' of ODN 1 and 32mer overhang at 5' of ODN 4	ODN 1 + ODN 4
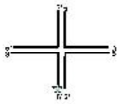 Holliday junction	Immobile Holliday junction with ~32 bp arms	ODN 1 + ODN 4 + ODN 10 + ODN 11
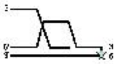 D-loop	Triplex DNA with ~30 mer loop in the middle	DL + DL-Forward + DL-Reverse
